# Disrupted neural correlates of anesthesia and sleep reveal early circuit dysfunctions in Alzheimer models

**DOI:** 10.1016/j.celrep.2021.110268

**Published:** 2022-01-18

**Authors:** Daniel Zarhin, Refaela Atsmon, Antonella Ruggiero, Halit Baeloha, Shiri Shoob, Oded Scharf, Leore R. Heim, Nadav Buchbinder, Ortal Shinikamin, Ilana Shapira, Boaz Styr, Gabriella Braun, Michal Harel, Anton Sheinin, Nitzan Geva, Yaniv Sela, Takashi Saito, Takaomi Saido, Tamar Geiger, Yuval Nir, Yaniv Ziv, Inna Slutsky

**Affiliations:** 1 Department of Physiology and Pharmacology, Sackler Faculty of Medicine, Tel Aviv University, Tel Aviv 69978, Israel; 2 Sagol School of Neuroscience, Tel Aviv University, Tel Aviv 69978, Israel; 3 Department of Neurobiology, Weizmann Institute of Science, Rehovot 76100, Israel; 4 Laboratory for Proteolytic Neuroscience, RIKEN Center for Brain Science, Saitama 351-0198, Japan; 5 Department of Neurocognitive Science, Institute of Brain Science, Nagoya City University Graduate School of Medical Sciences, Nagoya, Aichi 467-8601, Japan; 6 Department of Human Genetics and Biochemistry, Sackler Faculty of Medicine, Tel Aviv University, Tel Aviv 69978, Israel

**Keywords:** Alzheimer's disease, firing rate homeostasis, hippocampus, hyperexcitability, calcium imaging, single-unit recordings, general anesthesia, sleep, NREM, DHODH

## Abstract

Dysregulated homeostasis of neural activity has been hypothesized to drive Alzheimer’s disease (AD) pathogenesis. AD begins with a decades-long presymptomatic phase, but whether homeostatic mechanisms already begin failing during this silent phase is unknown. We show that before the onset of memory decline and sleep disturbances, familial AD (fAD) model mice display no deficits in CA1 mean firing rate (MFR) during active wakefulness. However, homeostatic down-regulation of CA1 MFR is disrupted during non-rapid eye movement (NREM) sleep and general anesthesia in fAD mouse models. The resultant hyperexcitability is attenuated by the mitochondrial dihydroorotate dehydrogenase (DHODH) enzyme inhibitor, which tunes MFR toward lower set-point values. *Ex vivo* fAD mutations impair downward MFR homeostasis, resulting in pathological MFR set points in response to anesthetic drug and inhibition blockade. Thus, firing rate dyshomeostasis of hippocampal circuits is masked during active wakefulness but surfaces during low-arousal brain states, representing an early failure of the silent disease stage.

## Highlights

• CA1 firing rates are similar between WT and fAD model mice in active wakefulness
• Down-regulation of CA1 firing rates by NREM sleep and anesthesia fails in fAD mice
• fAD mutations impair stabilization of lower firing rate set points by anesthetics
• DHODH inhibition suppresses CA1 hyperexcitability under anesthesia in fAD mice

## Graphical abstract

**Figure:**
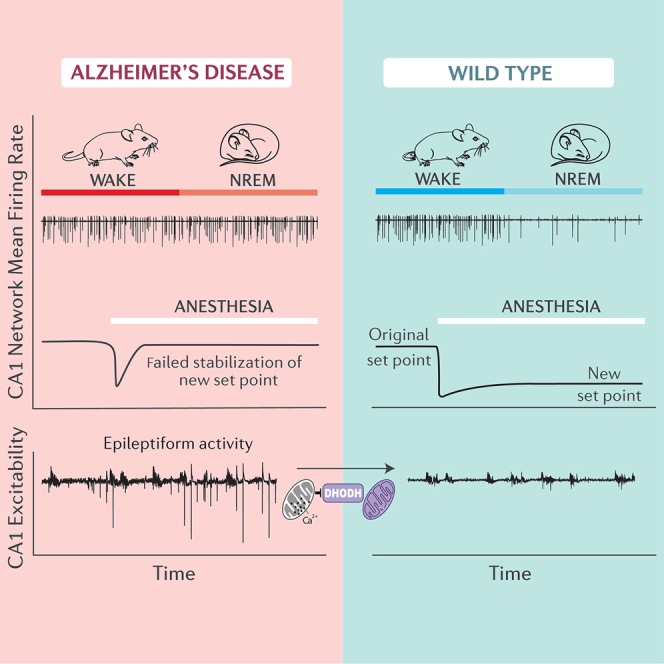

## Teaser

Zarhin et al. show that firing rate dyshomeostasis is masked during active wakefulness but surfaces during anesthesia and NREM sleep in hippocampal circuits, preceding global sleep and memory disturbances in a mouse model of familial Alzheimer’s disease.

## Introduction

Alzheimer’s disease (AD) is a progressive neurodegenerative disorder accounting for the vast majority of dementias. Hippocampal and cortical circuit dysfunctions are hypothesized to cause cognitive deficits in AD, such as episodic and spatial memory impairments. However, the onset of amyloid-β (Aβ) depositions precedes cognitive impairments by at least 10–20 years, marking a significant presymptomatic disease stage (Bateman et al., 2012; Long and Holtzman, 2019; Vermunt et al., 2019). By the time the earliest AD clinical symptoms are detectable, Aβ accumulation is close to reaching its peak, followed by intracellular aggregation of tau (Morris and Price, 2001). This suggests that homeostatic mechanisms successfully maintain critical aspects of neural circuits during early impairments of Aβ and tau proteostasis, but fail at some point, driving the emergence of the first symptoms (Frere and Slutsky, 2018; Styr and Slutsky, 2018). Identifying how circuit-level signatures are altered during the presymptomatic AD stage is crucial for understanding the transition from “silent” pathophysiology to clinically evident impairments.

Extensive experimental efforts over the past decades have identified the role of familial AD (fAD) mutations in early impairments of synaptic transmission and plasticity in hippocampal circuits via Aβ and other cleavage products of Aβ precursor protein (APP) processing (Fogel et al., 2014; Hsia et al., 1999; Li et al., 2009; Rice et al., 2019; Shankar et al., 2008; Wang et al., 2017; Willem et al., 2015). In addition to synaptic plasticity deficits, emerging evidence points to hyperexcitability of hippocampal and cortical neural networks in patients with amnestic mild cognitive impairment (Bakker et al., 2012), with AD (Horvath et al., 2021; Lam et al., 2017; Quiroz et al., 2010; Vossel et al., 2013, 2016), and in distinct AD mouse models (Busche et al., 2008, 2012; Hall et al., 2015; Minkeviciene et al., 2009; Palop et al., 2007; Palop and Mucke, 2009; Šišková et al., 2014; Verret et al., 2012; Zott et al., 2019).

Why is the activity of cortico-hippocampal circuits destabilized in early AD stages? It is hypothesized that homeostatic plasticity bidirectionally regulates neuronal activity around a stable set point to compensate for learning-related plasticity (Davis, 2006; Marder and Goaillard, 2006; Turrigiano and Nelson, 2004). Emerging evidence suggests that the distribution of firing rates among neurons in a neuronal circuit and their mean firing rate (MFR) are the key variables that are maintained around a set-point value in a process called firing rate homeostasis (Barnes et al., 2015; Hengen et al., 2013, 2016; Keck et al., 2013; Slomowitz et al., 2015; Styr et al., 2019). Our recent study suggests that homeostatic MFR set points are not predetermined but can be tuned by readjusting the compensatory feedback mechanisms to maintain a distinct MFR set-point value (Styr et al., 2019). Indeed, firing rate distributions and their means are physiologically regulated by sleep-wake states in distinct neural circuits, and this state-dependent regulation has been proposed to be homeostatic (Levenstein et al., 2017; Watson et al., 2016). MFRs decrease during sleep but return to higher MFR set points following transitions to active wakefulness in the rodent hippocampus and some areas of the cortex (Ju et al., 2014; Miyawaki and Diba, 2016; Niethard et al., 2021; Senzai et al., 2019; Vyazovskiy et al., 2009; Zhou et al., 2019). Other parameters of firing rate statistics are also regulated by sleep. For example, non-rapid eye movement (NREM) sleep, which makes up ∼80% of all sleep, is associated with homogenization of firing rate distributions, differentially regulating MFR of high-firing- and low-firing-rate neurons (Watson et al., 2016). This homeostatic regulation can operate at a local, layer-specificity scale in the cortex (Senzai et al., 2019). Thus, malfunction of state-dependent firing rate homeostasis in local neural circuits may be at the core of the early AD progression.

State-dependent changes in firing rates may be important for early progression of AD pathology. Aβ and tau soluble protein levels and aggregates are influenced by the sleep-wake cycle (Roh et al., 2012; Wang and Holtzman, 2020): their levels in the interstitial fluid are decreased by sleep and increased by sleep deprivation (Holth et al., 2019; Ju et al., 2017; Kang et al., 2009; Lucey et al., 2018; Xie et al., 2013). Furthermore, sleep is progressively deteriorated in patients with AD and in fAD mouse models, resulting in disrupted slow-wave activity (SWA) during NREM sleep, sleep fragmentation, and reduction in sleep time (Ju et al., 2017; Mander et al., 2015; Roh et al., 2012; Wang and Holtzman, 2020). We, therefore, hypothesized that local homeostatic dysregulation of MFRs in hippocampal circuits may take place before global changes in sleep become evident.

In addition to natural sleep, general anesthesia leads to a pronounced suppression of MFRs in non-human primates (Redinbaugh et al., 2020) and rodents (Suzuki and Larkum, 2020), as well as to a reduced number of discriminable neural activity patterns (Ferrarelli et al., 2010; Hudetz et al., 2015, 2020; Wenzel et al., 2019). Moreover, distinct general anesthetic drugs augment Aβ and tau soluble protein levels and their aggregation (Berger et al., 2016; Eckenhoff et al., 2004; Planel et al., 2007; Vutskits and Xie, 2016; Whittington et al., 2019; Xie et al., 2008; Zhang et al., 2013). Thus, in addition to sleep, general anesthesia may constitute a distinct low-arousal brain state that could also reveal early firing rate dyshomeostasis.

We combined large-scale *in vivo* Ca^2+^ imaging and electrophysiology to study the functional changes in the hippocampal circuits of fAD mouse models before the onset of cognitive decline. Our results demonstrate that CA1 neural dynamics and MFRs were preserved during active wakefulness but disrupted during NREM sleep and anesthesia, resulting in pathological CA1 hyperexcitability. In line with our hypothesis, this local dysregulation of CA1 MFRs precedes global disturbances in SWA during NREM sleep. Studying network-level firing rate homeostasis *ex vivo* suggests that fAD mutations disrupt the basic regulation of MFR set points by general anesthetic and homeostatic response to inhibition blockade, resulting in pathological MFR set points. Teriflunomide (TERI), an inhibitor of the mitochondrial enzyme dihydroorotate dehydrogenase (DHODH), a recently identified signaling pathway of MFR set-point down-regulation (Styr et al., 2019), suppressed CA1 hyperexcitability in anesthetized fAD mice. Overall, our study identifies a central role of low-arousal brain states in early vulnerability of hippocampal circuits in fAD models. Furthermore, it proposes that lowering firing rate set points during such states may present a new conceptual strategy for treating or preventing pathological hippocampal activity during the presymptomatic AD phase.

## Results

### CA1 population activity is normal during active wakefulness in APP/PS1 mice

First, we characterized amyloid homeostasis and hippocampus-dependent memory in 4- to 5-month-old APP/PS1 (APP_Swe_/PS1ΔE9) mice. These mice display pathologically increased Aβ40, Aβ42, and Aβ42/Aβ40 ratio for both soluble and insoluble Aβ fractions (Figures S1A–S1C). Hippocampus-dependent memory functions, such as spatial working memory and context-dependent fear memory, were unimpaired at this age (Figures S1D–S1F). Notably, memory decline was evident at a more advanced disease stage, in 9-month-old APP/PS1 mice (Figures S1G and S1H).

**Figure 1:**
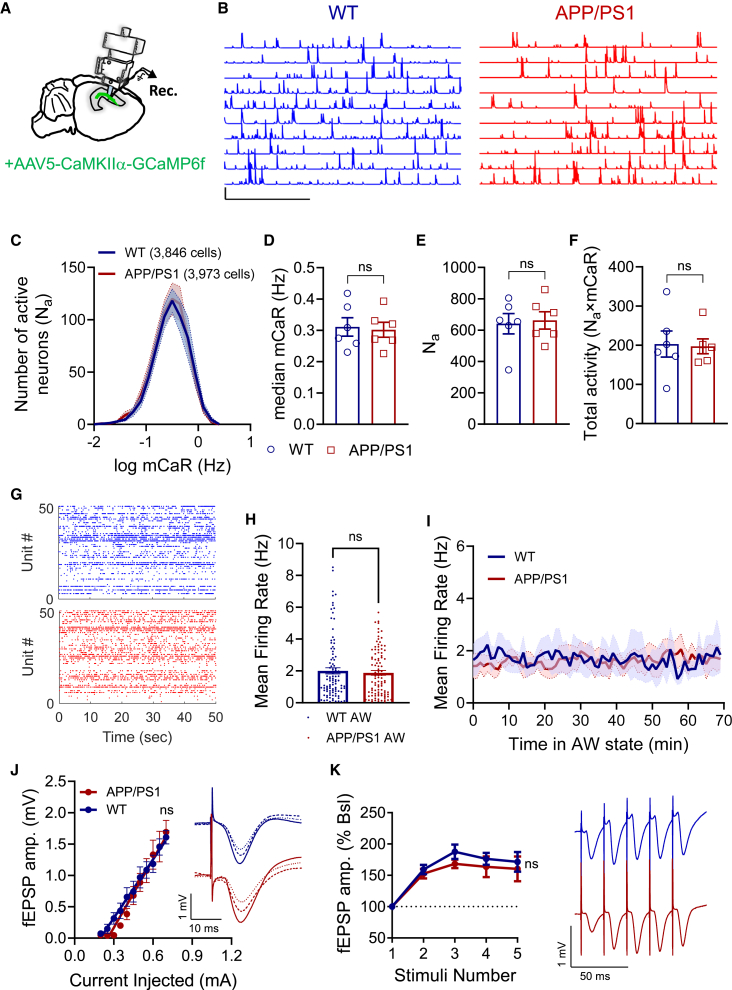
No deficits in the CA1 network activity and CA3-CA1 synaptic transmission during active wakefulness in APP/PS1 mice (A) Large-scale Ca^2+^ imaging of excitatory cells expressing GCaMP6f using wide-field, head-mounted miniaturized fluorescence microscope in freely behaving mice during exploration of familiar environment. (B) Relative fluorescence change traces of 10 cells randomly selected from the spatial locations depicted in Figure S3A, representing Ca^2+^ transients of cells detected in WT (left, blue traces) and APP/PS1 (right, red traces). Scale bars: 5 min; 10 *Z* scores. (C) Average mCaR distributions of CA1 neuronal populations in WT (6 mice, 3,846 cells) and APP/PS1 (6 mice, 3973 cells) during active wakefulness. (D–F) The median mCaRs of neurons (p = 0.94, D), number of N_a_s (p = 0.99, E), and total activity (mCaR^∗^N_a_, p = 0.82, F) were not different between WT and APP/PS1 mice (same data as in C, averaged per mouse) during active wakefulness. (G) Representative raster plots demonstrating CA1 single-unit spiking activity during active wakefulness in WT (blue) and APP/PS1 (red) mice. (H) MFRs of regularly spiking neurons during active wakefulness were not different (p = 0.82) between WT (2.01 ± 0.195; 4 mice, 103 single units) and APP/PS1 (1.88 ± 0.15; 4 mice, 98 single units) mice. (I) Dynamics of MFR change of regular spiking neurons across all active wakefulness episodes, concatenated (bold line represents the average MFR) in WT and APP/PS1 mice (same data as in H). Shaded area represents SEM. (J) No changes (p = 0.19) in CA3-CA1 basal synaptic transmission between awake WT (8 mice) and APP/PS1 (6 mice). Right: representative traces of fEPSPs of WT versus APP/PS1 evoked by 40, 50, and 60 μA stimulation. (K) No changes (p = 0.42) in short-term synaptic facilitation in response to 50-Hz burst between awake WT and APP/PS1 mice. Right: representative traces of fEPSPs evoked by five stimuli at 50 Hz. Unpaired Mann-Whitney nonparametric test (D–F and H). Two-way ANOVA with Sidak's multiple comparisons test (J and K) were used for the analysis. Error bars represent SEM. ns, non-significant. See also Figures S1–S3 and Videos S1 and S2.

To analyze neuronal activity in large neuronal populations in freely behaving mice, we employed wide-field, head-mounted miniaturized microscopes (Cai et al., 2016; Ghosh et al., 2011; Ziv et al., 2013) (Figures 1A and S2A–S2C). This technique enables tracking of Ca^2+^ dynamics with single-neuron resolution (Barretto et al., 2011; Ziv and Ghosh, 2015) as a proxy for neuronal activity (Chen et al., 2013). The integrated miniaturized microscope allows for high-speed and large-scale longitudinal recordings of Ca^2+^ dynamics from genetically defined neuronal populations in various deep brain structures, including the hippocampus, in freely behaving mice. Utilizing this method, we monitored activity patterns of thousands of CA1 pyramidal neurons and analyzed active neurons (N_a_s) at each session during active wakefulness. We imaged fluorescence generated by a genetically encoded Ca^2+^ sensor GCaMP6f (Chen et al., 2013) expressed in excitatory CA1 pyramidal neurons under the Ca^2+^/calmodulin-dependent protein kinase IIα (CaMKIIα) promoter. Experiments were performed in six APP/PS1 mice (3,973 neurons total) and six wild-type (WT) littermates (3,846 neurons total), while they explored a familiar open field. Imaging was performed daily at regular hours during light phase to avoid circadian effects. Firing rate of single cells was approximated from the Ca^2+^ event rates using the CMNF-E method (Zhou et al., 2018) optimized for one-photon Ca^2+^ imaging (Figures 1B, 1C, and S2D–S2G). No detectable changes in Ca^2+^ event rate distributions of CA1 neuronal populations were found between WT and APP/PS1 mice in active wakefulness (Figures 1B and 1C; Videos S1 and S2). Mean Ca^2+^ event rate (mCaR) per cell was very heterogeneous in both WT and APP/PS1 mice (Figure 1C). No differences were observed in physical activity of WT versus APP/PS1 mice during exploration of a familiar environment (Figures S3A–S3E). Detailed analysis of all recorded neurons revealed no difference in the median mCaR (Figure 1D) or in the number of N_a_s (Figure 1E), resulting in similar total level (N_a_^∗^mCaR) of CA1 activity between WT and APP/PS1 mice (Figure 1F). Also, no differences were detected in the statistical attributes of CA1 network activity between behaving WT and APP/PS1 mice (Figures S3F–S3H).

Video S1. Calcium dynamics in soma of CA1 excitatory neurons in WT mouse exploring familiar environment, related to Figure 1 Presented are the Ca^2+^ imaging microendoscopic raw data (upper left), CNMF-E denoised and scaled neural spatial distributions and Ca^2+^ transients (upper right) and the corresponding raster plots of Ca^2+^ transients from the same cells detected by CNMF-E algorithm. White arrow marks the presented frame.

Video S2. Calcium dynamics in soma of CA1 excitatory neurons in APP/PS1 mouse exploring familiar environment, related to Figure 1 Presented are the Ca^2+^ imaging microendoscopic raw data (upper left), CNMF-E denoised and scaled neural spatial distributions and Ca^2+^ transients (upper right) and the corresponding raster plots of Ca^2+^ transients from the same cells detected by CNMF-E algorithm. White arrow marks the presented frame.

Although somatic Ca^2+^ signals in neurons are used as a proxy of spiking activity, whether spike-to-Ca^2+^ transfer function is preserved under variable experimental conditions is unknown, and the sensitivity of microendoscopy for Ca^2+^ transients evoked by a single action potential remains to be improved. Therefore, we used chronically implanted tetrodes to directly record single-unit spiking activity in behaving WT and APP/PS1 mice. The recordings of CA1 firing rates were performed during the same hours of light phase as Ca^2+^ recordings, in a familiar environment (home cage). Vigilance state analysis was performed by analyzing local field potential (LFP) and electromyogram (EMG) in the same mice with single-unit recordings (Figures S6A and S6B) and in a separate batch of mice by analyzing electroencephalogram (EEG)/EMG and video recordings (Figure S4). Criteria for clustering of single units (Figures S5A–S5C) and separation of regular spiking (RS), putative pyramidal neurons from fast-spiking (FS), putative interneurons (Figures S5D–S5F) were based on a previous analysis (Petersen et al., 2021) and optimized for our recording conditions. Our results show no difference (p = 0.82) in MFR of the CA1 population of RS neurons between WT (2.01 ± 0.195 Hz) and APP/PS1 mice (1.88 ± 0.15 Hz) in active wakefulness (Figures 1G–1I). Furthermore, no difference was observed in MFRs of CA1 FS neurons (Figure S5G, p = 0.22). The duration of active wake state (Figure S6C) and physical activity of mice (Figures S6D–S6E) were similar between groups. Thus, two independent recording methods with single-neuron resolution, Ca^2+^ microendoscopy and single-unit electrophysiology, demonstrate that CA1 MFRs are unaltered during active wakefulness in early-stage APP/PS1 mice.

Finally, we recorded the extracellular field excitatory postsynaptic potentials (fEPSPs) in the CA3-CA1 pathway in awake WT and APP/PS1 mice. No changes were observed in CA3-CA1 synaptic transmission and short-term synaptic plasticity between awake WT and APP/PS1 mice (Figures 1J and 1K). Overall, these results demonstrate that CA1 firing rates, CA3-CA1 synaptic transmission, and short-term plasticity are not impaired during active wakefulness in APP/PS1 mice before the onset of memory decline.

### Dysregulation of CA1 MFRs during NREM sleep in APP/PS1 mice

**Figure 2:**
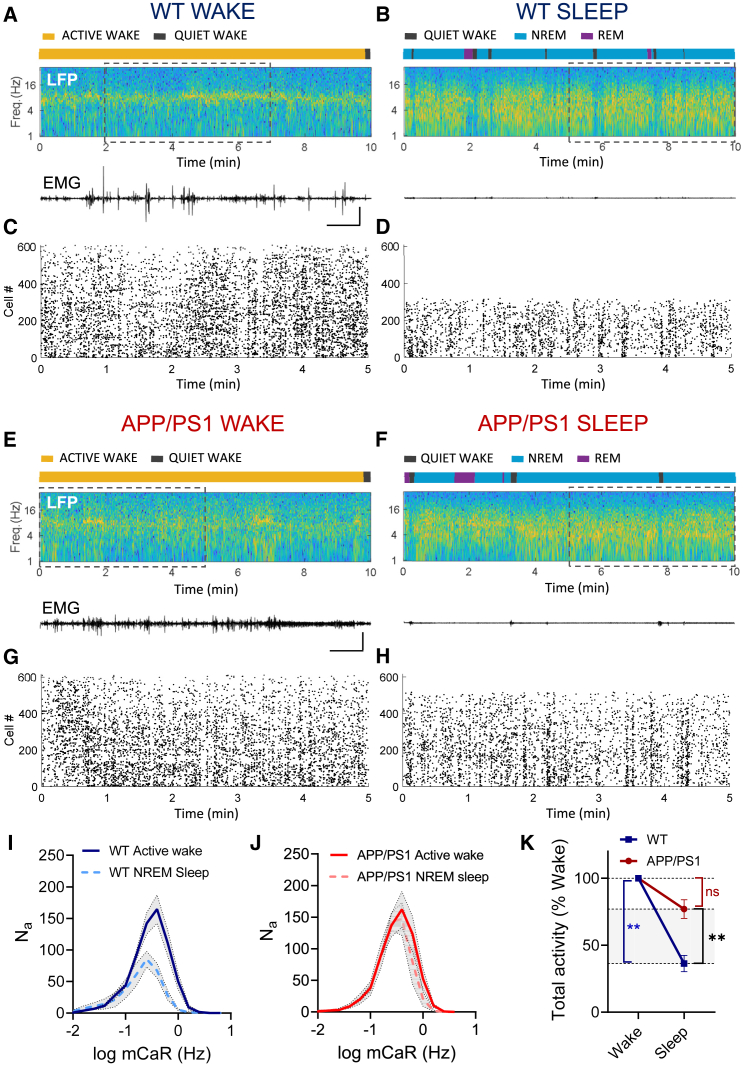
Reduction of CA1 population activity during NREM sleep is impaired in APP/PS1 mice (A and B) An example of wake-dense (A) and sleep-dense (B) recordings from a WT mouse. Top: hypnograms, generated by manual brain state segregation. Brain states are color coded: yellow, active wake; gray, quiet wake; blue, NREM sleep; purple, REM sleep. Middle: Fourier transform-based LFP power spectrograms. Bottom: EMG traces (scale bars: 0.5 mV, 1 min). (C and D) Representative raster plots of CA1 Ca^2+^ event rates in active wake-dense episode (C) and NREM-dense episode (D) in a WT mouse (time interval corresponds to dashed rectangles in A and B). (E–H) Same as (A)–(D) for APP/PS1 mice. (I) Average mCaR distributions in CA1 of WT mice (5 mice) during active wake (3,319 cells) and NREM sleep (1,741 cells) states. (J) Average mCaR distributions in CA1 of APP/PS1 mice (4 mice) during active wake (2,595 cells) and NREM sleep (2,224 cells) states. (K) Relative change in total activity in WT versus APP/PS1 mice by NREM sleep in comparison to active wake state (the same data as in I and J). Two-way ANOVA with Sidak's multiple comparisons test (K, inter-group analysis); two-way ANOVA (K, intra-group analysis). ^∗∗^p < 0.01. Error bars represent SEM. See also Figures S4A–S4C.

Next, we asked whether local homeostatic mechanisms underlying down-regulation of CA1 neuronal activity by NREM sleep (Miyawaki and Diba, 2016; Zhou et al., 2019) are maintained in the early-stage fAD mice. To analyze regulation of CA1 neuronal activity by sleep, we imaged CA1 dynamics, in parallel with LFP/EMG recordings, during the sleep-wake cycle of mice. The recordings were performed in five WT and four APP/PS1 mice during the same hours of the light cycle. Ca^2+^ imaging was analyzed during periods of wake-dense episodes dominated by active wakefulness (Figures 2A and 2E) and sleep-dense episodes dominated by NREM sleep periods (Figures 2B and 2F). As expected, WT mice showed ∼60% reduction in total CA1 activity during NREM sleep because of a reduction in the number of N_a_s and the mCaR, in comparison to active wakefulness (Figures 2A–2D and 2I). In contrast to WT mice, neither the number of N_a_s nor the mCaR was significantly changed during NREM sleep of APP/PS1 mice, resulting in similar total activity levels during active wake and NREM sleep periods (Figures 2E–2H and 2J). Thus, the typical negative regulation of CA1 population activity by NREM sleep was significantly diminished in APP/PS1 in comparison to WT mice (Figure 2K). These results indicate that homeostatic regulation of CA1 MFRs is impaired in a state-dependent manner in APP/PS1 mice.

### Local dysregulation of CA1 firing rates during NREM sleep precedes global deterioration of SWA in APP/PS1 mice

**Figure 3:**
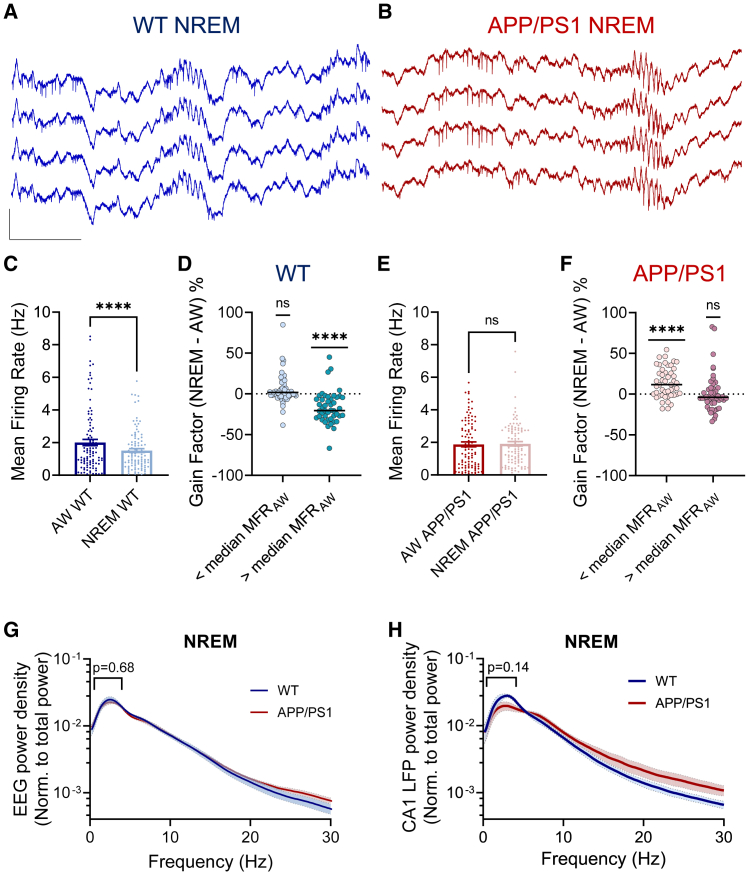
Local dysregulation of CA1 firing rates by NREM sleep precedes global SWA deterioration in APP/PS1 mice (A and B) Raw representative traces from a tetrode in WT (A) and APP/PS1 (B) mice during NREM sleep. Scale bars: 0.5 mV, 100 ms. (C) NREM sleep causes a reduction (p < 0.0001) in CA1 MFR of RS neurons from 2.01 ± 0.195 Hz in active wakefulness (AW) to 1.51 ± 0.12 Hz in WT mice (4 mice, 103 single units). (D) WT neurons whose MFRs were below the median firing rate during AW (1.4 Hz) showed no change in the gain factor in NREM sleep (+1.7%, confidence interval [CI] = [−0.8%, 3.1%]; p = 0.055), while cells whose MFRs were above the median showed a decrease in gain factor of −21% in NREM sleep (CI = [−27%, −11%], p < 0.0001) during sleep (same data as in C). (E) MFR of CA1 RS neurons is not different (p = 0.53) between AW (1.88 ± 0.15 Hz) and NREM sleep (1.91 ± 0.13 Hz) in APP/PS1 mice (4 mice, 98 units). (F) In APP/PS1 mice, cells whose MFRs were below the median firing rate during AW showed an increase of MFR in NREM sleep with median gain factor +12% (CI = [2.7%, 22%]; p < 0.0001), while cells whose MFRs were above the median showed no change in NREM sleep (−4%, CI = [−5.8%, 1.4%]; p = 0.22, same data as in E). (G) Frontal EEG spectra during NREM sleep in WT (n = 5) and APP/PS1 (n = 5) mice. Post hoc comparisons did not reveal significant genotype differences for any of the SWA frequency bins in NREM state (p = 0.68). (H) CA1 LFP spectra recorded by the same electrodes as single units in WT (n = 4) and APP/PS1 (n = 4) mice during NREM sleep. Slow-wave power during NREM is not significantly different between WT and APP/PS1 mice (p = 0.14). Non-parametric, paired, two-tailed Wilcoxon test (C and E), two-way ANOVA with Sidak's multiple comparisons test (G and H), one-sample Wilcoxon test comparing medians of the samples to 100% (D and F). ^∗∗∗∗^p < 0.0001. Error bars represent SEM. See also Figures S4D–S4G, S5, and S6.

Next, we asked whether local homeostatic regulation of firing rates by NREM sleep is impaired because of the deterioration of slow-wave oscillations, as reported in patients with AD (Lucey et al., 2019; Mander et al., 2015) and in fAD mouse models after the onset of cognitive decline (Kent et al., 2018). To compare CA1 firing rates between WT and APP/PS1 mice, we used single-unit recordings (Figures 3A and 3B). In WT mice, MFRs of CA1 RS neurons were decreased on average from 2.01 ± 0.195 to 1.51 ± 0.12 Hz during NREM sleep in comparison to active wakefulness (Figure 3C). We proceeded to quantitatively compare MFR of each unit in active wakefulness and NREM sleep across the entire dataset. A sub-population of CA1 high-rate excitatory neurons (defined as neurons whose MFRs during active wakefulness were above the median of 1.4 Hz) showed attenuation with median gain factor of −21% in NREM sleep (45% of all the units, p < 0.0001; Figure 3D). However, a sub-population of low-firing neurons (defined as neurons whose MFRs were below the median) displayed heterogeneous gain factors with a tendency toward a small increase (p = 0.055; Figure 3D), confirming the results of a previous study conducted in the cortex (Watson et al., 2016). This physiological regulation of firing rates in CA1 pyramidal neurons by NREM sleep was disrupted in APP/PS1 mice. On average, MFRs of the CA1 network were not different (1.88 ± 0.15 Hz in AW versus 1.91 ± 0.13 Hz in NREM; p = 0.53) between active wakefulness and NREM sleep (Figure 3E). The loss of negative regulation of the network MFRs by NREM sleep was due to the reconfiguration of MFRs within the local CA1 network. Namely, low-firing-rate units showed an increase in NREM sleep with median gain factor of +12% (p < 0.0001, 50% of all the units; Figure 3F), while the gain factor of high-firing-rate units was not affected by NREM sleep (p = 0.28; Figure 3F). Thus, fAD mutations augmented a positive effect of NREM sleep on low-firing-rate neurons, but diminished its negative effect on high-firing-rate neurons. FS neurons in the CA1 showed a similar regulation by NREM sleep: MFR reduction was prominent in WT but lost in APP/PS1 mice (Figures S5H and S5I). The duration of active wake and NREM sleep states was not different between the genotypes (Figure S6C).

Because global SWA is known to be disrupted in patients with AD (Lucey et al., 2019; Mander et al., 2015) and in fAD mouse models after the onset of cognitive decline (Kent et al., 2018), we used EEG/EMG recordings (Figures S4A–S4C) to test how global SWA is affected in APP/PS1 mice before robust cognitive impairments are evident. No differences in EEG SWA (spectral power of 0.5–4 Hz) during NREM sleep were found between WT and APP/PS1 mice (Figure 3G). Furthermore, EEG power spectra were not different for other vigilance states, as well as between WT and APP/PS1 mice (Figures S4D–S4F). In contrast to the similar global SWA in 4- to 5-month-old mice, EEG SWA significantly decreased in older 9-month-old APP/PS1 mice showing memory decline (p = 0.002; Figure S4G), as expected (Kent et al., 2018). Notably, the LFP SWA measured by tetrodes in the local CA1 circuitry showed a tendency toward lower levels during NREM sleep in 4- to 5-month-old APP/PS1 mice but did not reach statistical significance (p = 0.14; Figure 3H). This non-significant trend for lower SWA in CA1 LFPs represents an intermediate scale between the observed differences at the local single-neuron level and the lack of differences at the global EEG scale.

Taken together, Ca^2+^ imaging and electrophysiological data with single-cell resolution suggest that local down-regulation of CA1 MFRs by NREM sleep is disrupted in early-stage APP/PS1 mice, and this homeostatic dysregulation of firing rates precedes global deterioration of slow-wave oscillations.

### Loss of neuronal suppression during general anesthesia in APP/PS1 mice

**Figure 4:**
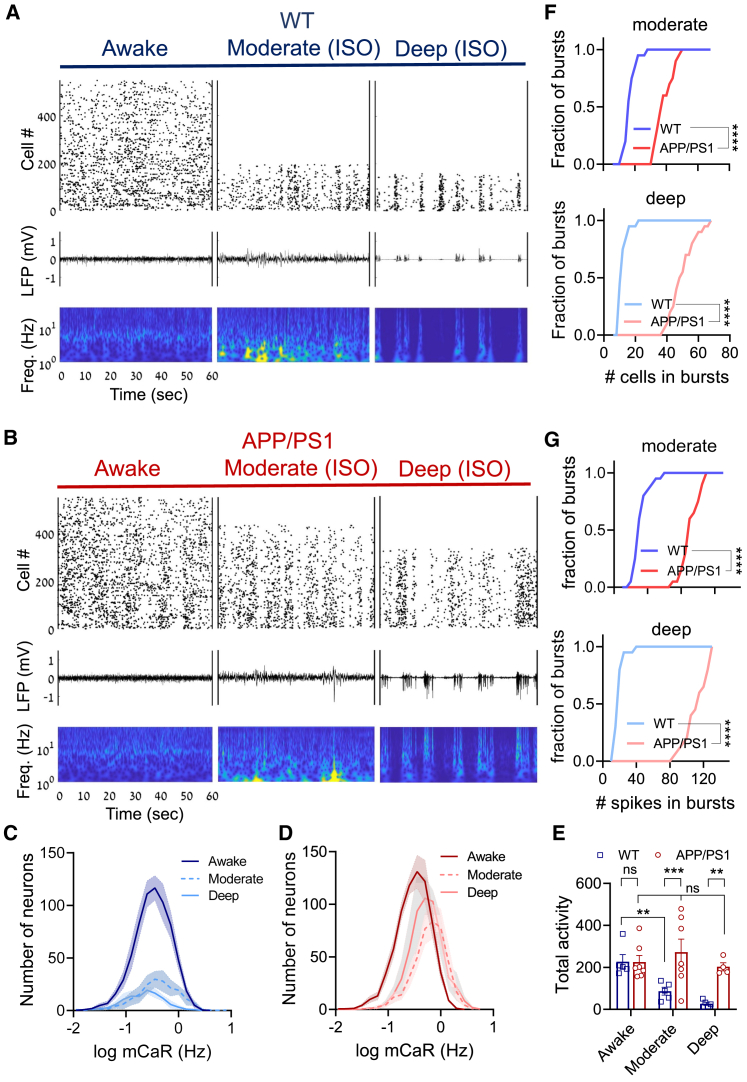
Loss of neuronal inhibition across anesthetic depth in APP/PS1 mice (A and B) Representative traces demonstrating raster plots of Ca^2+^ transients (top), raw LFP (middle), and corresponding scalograms based on wavelet transformation (bottom, log scale) for both WT (A) and APP/PS1 (B) under exploration in familiar environment (active wake), moderate anesthesia (moderate), and under deep anesthesia (deep). Imaging was performed in parallel to CA1 LFP recordings. (C and D) Average mCaR distribution of CA1 neuronal populations in WT (C, 5 mice per condition) and APP/PS1 (D, awake and moderate: 7 mice; deep: 4 mice) under all three conditions described in (A) and (B). Note higher mCaR and number of active neurons in anesthetized APP/PS1 versus WT mice. (E) Total activity is higher in APP/PS1 versus WT mice under both moderate and deep anesthesia (same data as in C and D). (F and G) Differences in temporal patterns of Ca^2+^ transients in CA1 network of WT versus APP/PS1 anesthetized mice. APP/PS1 mice display a larger number of cells that participate in network burst (F, p = 0.0001 for moderate [top] and deep [bottom]) and a larger number of spikes that constitute a network burst (G, p = 0.0001 for moderate [top] and deep [bottom]). Two-way-ANOVA with Sidak's multiple comparisons test (E, inter-group analysis), two-way ANOVA with Tukey's multiple comparison test (F and G), and one-way ANOVA with Dunnett's multiple comparisons test (E, intra-group analysis) were used for the analysis. ^∗∗^p < 0.01; ^∗∗∗^p < 0.001; ^∗∗∗∗^p < 0.0001; ^ns^p > 0.05. Error bars represent SEM. See also Figures S7–S9 and Videos S3 and S4.

We next asked whether APP/PS1 mice also display dysregulated CA1 activity under a distinct low-arousal state, such as general anesthesia. We first used the volatile gas anesthetic isoflurane because of its fast kinetics. We assessed three consecutive conditions associated with distinct LFP patterns (Figures 4A and 4B; Figures S7A–S7C): exploration in familiar environment in active wakefulness (low-amplitude high-frequency activity), moderate anesthesia (1% isoflurane, high delta 1–4 Hz power, sporadic responses to tail pinching), and deep anesthesia (1.5% isoflurane, burst suppression, unresponsiveness to tail pinching). Importantly, electrophysiological markers of anesthetic depth (Figure S7D) and respiratory rate (Figures S7E and S7F) were closely monitored and showed no differences between anesthetized WT and APP/PS1 mice, ruling out altered respiration or pharmacokinetics as potential factors.

In WT mice, isoflurane caused a pronounced inhibition of CA1 population activity (Figures 4A and 4C; Video S3). The distribution of mCaRs across cells revealed suppression of total CA1 activity by ∼57% and ∼87% during moderate and deep anesthesia, respectively (Figures 4C and 4E), mainly because of reduction in the number of N_a_s in WT mice (Figure 4C). General anesthesia had a different effect on CA1 population activity in APP/PS1 mice when compared to WT mice. Both moderate and deep anesthesia states expressed a reduced number of N_a_s that was accompanied by an increase in mCaR (p < 0.05, right shift in the distribution; Figures 4B and 4D). As a result, total activity level was maintained across wakefulness and anesthesia (Figures 4D and 4E; Video S4). The loss of a typical response to general anesthetic resulted in CA1 hyperactivity in anesthetized APP/PS1 relative to WT mice (Figure 4E). Moreover, the number of discriminable micro-patterns of activity (microstates) was also reduced under anesthesia in WT, but not in APP/PS1, mice (Figure S8). CA1 population activity became hyper-synchronized in anesthetized APP/PS1 mice, in comparison to WTs, as reflected by more neurons participating in network bursts (Figure 4F) and more spikes evoked per network bursts (Figure 4G). In addition, anesthetized APP/PS1 mice displayed an augmented input-output slope at CA3-CA1 synaptic connections, in comparison to WT mice (Figures S9A and S9B). Thus, both basal CA3-CA1 synaptic transmission and spontaneous CA1 spiking activity were selectively impaired in anesthesia, but not in active wakefulness, in APP/PS1 mice.

Video S3. Neuronal CA1 network decreases after deep isoflurane anesthesia in WT mouse, related to Figure 4 Calcium activity in soma of excitatory neurons after stabilization of burst-suppression rate. Presented are the Ca^2+^ imaging microendoscopic raw data (upper left), CNMF-E denoised and scaled neural spatial distributions and Ca^2+^ transients (upper right) and the corresponding raster plots of Ca^2+^ transients from the same cells detected by CNMF-E algorithm. White arrow marks the presented frame.

Video S4. Suppression of CA1 neuronal activity by isoflurane is lost in APP/PS1 mice, related to Figure 4 Calcium activity in soma of excitatory neurons after stabilization of burst-suppression rate. Presented are the Ca^2+^ imaging microendoscopic raw data (upper left), CNMF-E denoised and scaled neural spatial distributions and Ca^2+^ transients (upper right) and the corresponding raster plots of Ca^2+^ transients from the same cells detected by CNMF-E algorithm. White arrow marks the presented frame.

To test whether the aberrant activity of CA1 neurons in APP/PS1 mice is specific to isoflurane or a general feature of anesthesia, we recorded CA1 activity under ketamine-xylazine (KX) anesthesia. Ketamine is a dissociative anesthetic, inducing anesthesia mainly through blockade of *N*-methyl-d-aspartate (NMDA)-type glutamate receptors (Franks, 2008), and is often supplemented with xylazine, an α_2_-adrenergic receptor agonist. In WT mice, KX anesthesia resulted in a profound reduction in CA1 activity but was much less efficient in APP/PS1 mice (Figures S9C–S9E). Finally, medetomidine (MED), another general anesthetic that selectively activates α_2_-adrenergic receptors, caused pronounced inhibition of CA1 activity in WT mice, while a much lower level of suppression was observed in APP/PS1 mice (Figures S9F–S9H). Collectively, our results indicate that APP/PS1 mice express abnormal profiles of neural activity induced by several distinct classes of anesthetics, and that such changes are not due to a specific anesthetic drug.

### Distinct fAD models express CA1 hyperexcitability during anesthesia

**Figure 5:**
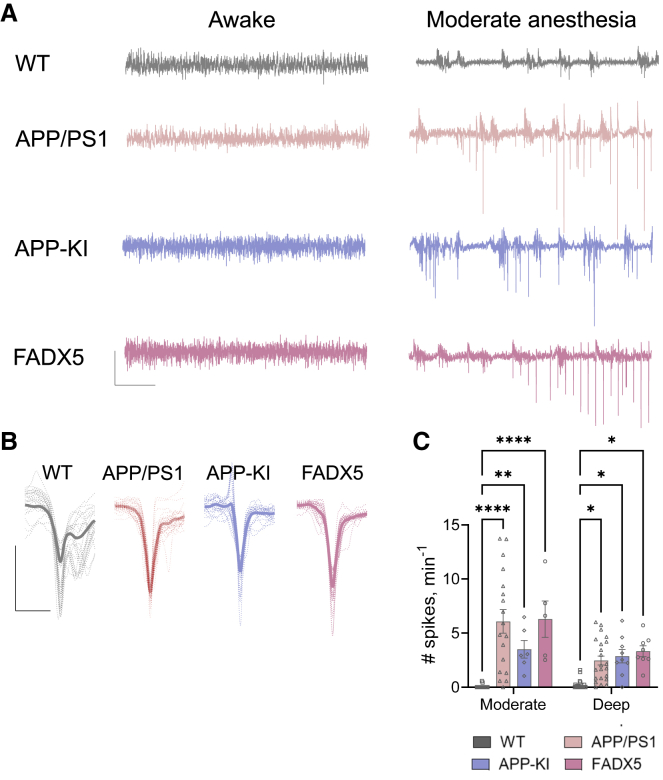
CA1 is hyperexcitable under anesthesia in different fAD models (A) Representative traces of raw CA1 LFP recordings during awake behavior (left) and moderate isoflurane anesthesia (right). Notice the appearance of abnormal spikes under anesthesia. LFP recordings were performed across different mouse fAD models: APP/PS1, 5XFAD, and APP-KI. Scale bars: 10 s, 1 mV. (B) Representative epileptiform spikes detected during moderate anesthesia across distinct fAD models. Scale bars: 50 ms, 1 mV. (C) On average, all three fAD models displayed higher frequency of epileptiform spikes compared to WT across isoflurane-induced moderate (WT: 17 mice, APP/PS1: 18 mice, FADX5: 5 mice, APP-KI: 6 mice) and deep (WT: 22 mice, APP/PS1: 22 mice, FADX5: 8 mice, APP-KI: 9 mice) anesthesia. Two-way-ANOVA with Dunnett's multiple comparisons test (C) was used for the analysis. ^∗^p < 0.05; ^∗∗^p < 0.01; ^∗∗∗∗^p < 0.0001. Error bars represent SEM. See also Figures S7E and S7F and Videos S5 and S6.

Augmented and hyper-synchronous Ca^2+^ dynamics in the soma of excitatory CA1 neurons of anesthetized fAD model mice prompted us to investigate whether these mice express silent epileptiform spikes, similarly to those detected in sleeping patients with AD (Lam et al., 2017). To address this question, we tested how general anesthesia affects CA1 network excitability using *in vivo* electrophysiological recordings in the CA1 *stratum radiatum* across different mouse fAD models. In addition to the APP/PS1 model, we made measurements in 5XFAD (Oakley et al., 2006) and *App*^*NL-G-F*^ knockin model (Saito et al., 2014) (APP-KI). APP-KI mice express physiological APP levels (Saito et al., 2014) but demonstrate an increased Aβ42/Aβ40 ratio (Figures S1A–S1C). In brief, epileptiform high-voltage spikes were detected across all fAD models but rarely in WT mice during isoflurane anesthesia (Figures 5A and 5B). To further understand the relationship between anesthesia states and CA1 hyperexcitability, we quantified the frequency of abnormal spiking activity across anesthetics depth. State-specific analysis revealed higher epileptiform spike frequency for all three fAD models compared to WT, for both moderate and deep anesthesia (Figure 5C; Videos S5 and S6). Importantly, respiration rate showed no differences between all three fAD models and WT anesthetized mice at both moderate and deep anesthesia (Figures S7E and S7F). These results indicate that pathological CA1 hyperexcitability is a common neuronal network dysfunction that emerges during low-arousal states. Moreover, the robustness of anesthesia-induced CA1 hyperexcitability in APP-KI mice indicates that fAD mutations on their own, even without APP overexpression, are sufficient to cause CA1 hyperexcitability under anesthetic-induced alerted states of arousal.

Video S5. An example of LFP recording in CA1 stratum radiatum in WT mouse under deep isoflurane anesthesia, related to Figure 5

Video S6. An example of LFP recording in CA1 stratum radiatum in APP/PS1 mouse under deep isoflurane anesthesia, related to Figure 5

### Downward firing rate homeostasis is impaired by fAD mutations

**Figure 6:**
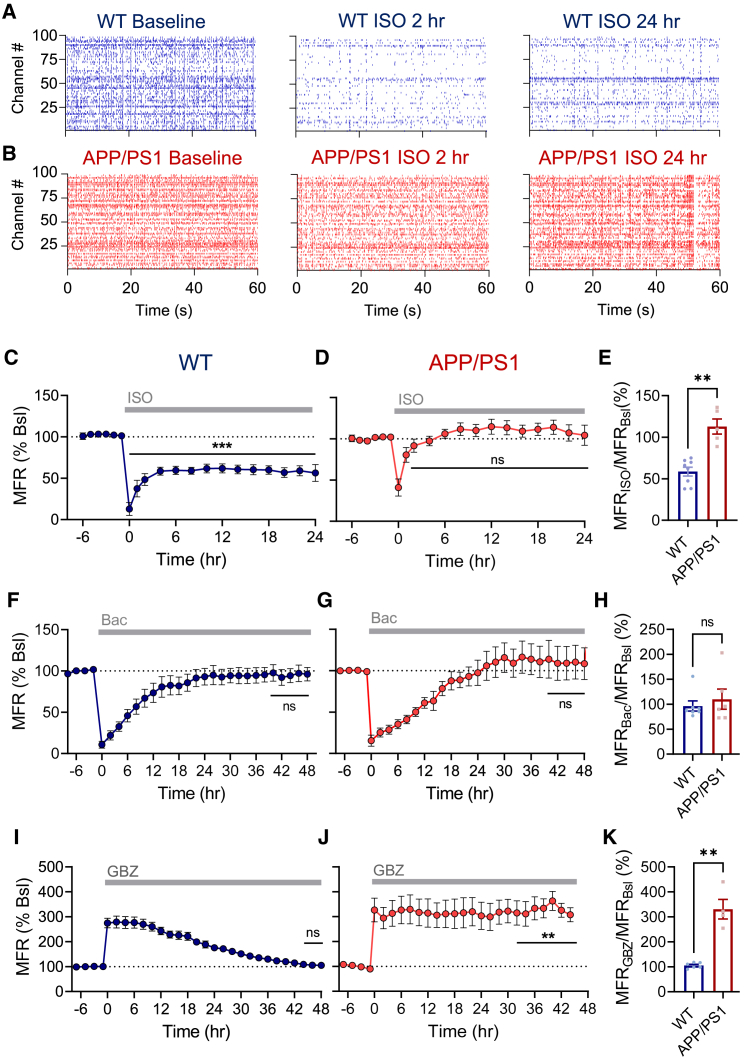
Dysregulation of downward MFR homeostasis by fAD mutations in hippocampal networks *ex vivo* (A and B) Raster plots from MEA recordings showing activity of the same channels in baseline, after 2 and 24 h of isoflurane (1%, 40mL/min) application in WT (A, blue, 97 channels) and APP/PS1 (B, red, 99 channels) cultures. (C) The MFR of the network was stably reduced after isoflurane (ISO) application to 42.0% ± 0.9% of baseline in WT cultures (n = 8 experiments, 633 channels). (D) The MFR of the network was decreased and rapidly compensated to 112.5% ± 8.7% (p > 0.9) of baseline after ISO application in APP/PS1 cultures (n = 5 experiments, 384 channels). (E) Summary of isoflurane (24 h) effect on MFRs in WT and APP/PS1 cultures (same data as C and D). (F and G) A typical MFR homeostatic response to chronic inactivity induced by a GABA_B_ receptor agonist baclofen (10 μM) in WT (F) and APP/PS1 (G) networks. MFR was homeostatically compensated during the first day of the perturbation to the MFR set-point level in WT (7 experiments, 394 channels, p = 0.76) and APP/PS1 (n = 6, p = 0.64, 377 channels) neurons. (H) Summary of baclofen (48 h) effect on MFRs in WT and APP/PS1 cultures (same data as F and G). (I and J) MFR was renormalized to the baseline level following 2 days of gabazine (GBZ, 30 μM) application in WT neurons (p = 0.31, n = 6 experiments, 398 channels), but MFR renormalization to GBZ was disrupted in APP/PS1 neurons (n = 4 experiments, 270 channels). (K) Summary of gabazine (48 h) effect on MFRs in WT and APP/PS1 cultures (same data as I and J). Paired two-tailed t test between the baseline and the perturbation (C, D, F, G, I, and J) and Mann-Whitney nonparametric two-tailed test (E, H, and K) were used for the analysis. ^∗^p < 0.05, ^∗∗^p < 0.01, ^∗∗∗^p < 0.001. The error bars represent SEM. See also Figures S10A and S10B.

The exposure of CA1 hyperexcitability under general anesthesia may be caused by impaired homeostatic regulation of MFRs. Hippocampal networks grown *ex vivo* on multi-electrode arrays (MEAs) have been previously established as an excellent model for dissecting the mechanisms of MFR homeostasis (Slomowitz et al., 2015; Styr et al., 2019). Therefore, we used this *ex vivo* platform to address the role of fAD mutations in firing rate homeostasis in response to the general anesthetic isoflurane. Spontaneous spiking activity of cultured hippocampal neurons grown on 120-channel MEAs was continuously monitored in an incubator chamber during a baseline recording period and for 24 h following application of isoflurane. Infusion of isoflurane (1%, 40 mL/min) stably reduced MFR in WT neurons without inducing a compensatory response (Figures 6A and 6C). This is in striking contrast to a typical MFR renormalization to the baseline level during 1 day in response to inactivity, induced by GABA_B_ receptor agonist baclofen as an example (Figure 6F) (Slomowitz et al., 2015). The observed compensatory response to decreased spiking activity confirms the idea that homeostatic mechanisms maintain stable circuit function by keeping network MFR around a set point. Moreover, our data demonstrate that this effect is not specific to isoflurane but is characteristic of structurally distinct anesthetics (Figures S10A and S10B). These results indicate that anesthetic drugs disable compensatory homeostatic mechanisms; thus, they constitute negative regulators of MFR set points. In contrast to the persistent suppression of MFRs in WT networks, isoflurane induced smaller and transient MFR decrease in APP/PS1 networks, accompanied by a fast MFR compensation to the original set point (Figures 6B and 6D). As a result, significant hyperexcitability was observed following isoflurane application in APP/PS1 in comparison to WT networks (Figure 6E). Notably, fAD mutations did not significantly impair homeostatic MFR response to inactivity (Figures 6G and 6H). These results suggest that fAD mutations disrupt basic homeostatic regulation of MFR set points by isoflurane.

Because isoflurane and other volatile anesthetics are known to augment inhibition by prolonging inhibitory currents mediated by GABA_A_ receptors (GABA_A_Rs) in hippocampal neurons (Jones and Harrison, 1993), among other targets, we decided to test how fAD mutations affect the homeostatic response to hyperactivity imposed by GABA_A_R blockade. As expected (Vertkin et al., 2015), application of a GABA_A_R antagonist gabazine (30 μM) caused a fast and pronounced increase in the population firing rate that gradually declined within 2 days to the set-point level (Figure 6I), despite the constant presence of gabazine. However, fAD mutations impaired MFR compensation in response to GABA_A_R blockade (Figure 6J), resulting in pathologically high MFR set points in APP/PS1, in comparison to WT networks (Figure 6K). Taken together, these results demonstrate that fAD mutations result in severe impairments of homeostatic MFR regulation, and these deficits can be uncovered even in 3-week-old cultured hippocampal neurons in the dish, disconnected from other brain structures. Specifically, fAD mutations dysregulate the downward homeostatic mechanisms that normally keep lower MFR set points by general anesthetics or return MFRs from hyperactivity.

### DHODH inhibition suppresses CA1 hyperexcitability under anesthesia

**Figure 7:**
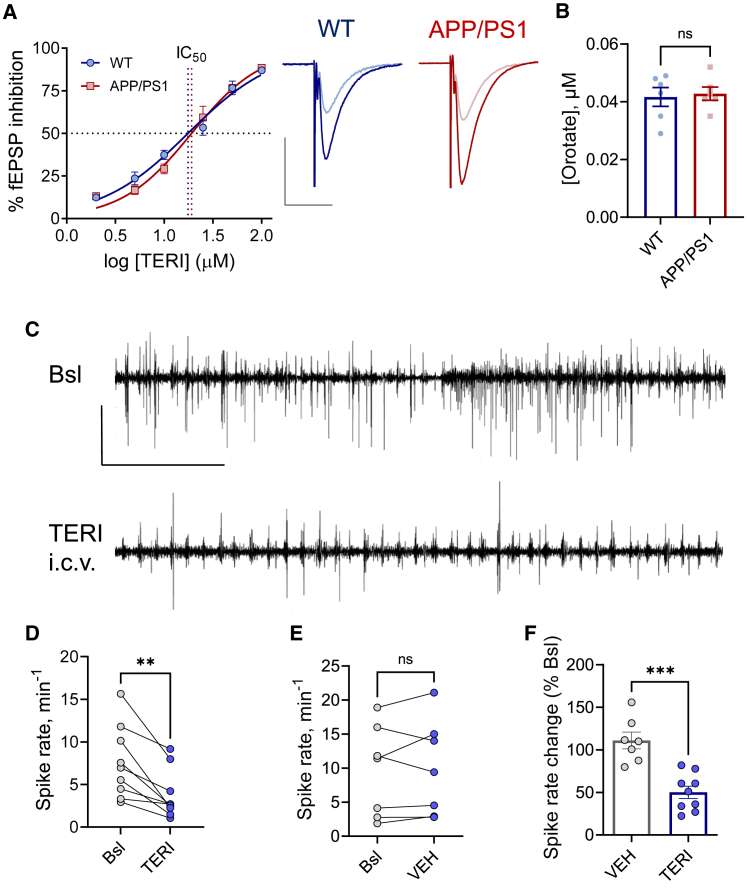
Teriflunomide reduces CA1 hyperexcitability in anesthetized APP/PS1 mice (A) Dose-response effect of TERI on the fEPSP amplitude in CA3-CA1 synaptic connections in hippocampal slices of WT (8 mice, 13 slices) and APP/PS1 (8 mice, 13 slices). IC_50_: 17.6 ± 1 μM in WT, 19.2 ± 1 μM in APP/PS1. Right: representative fEPSP traces before (dark blue/red) and after 25 μM TERI (light blue/red). Scale bars: 0.5 mV, 20 ms. (B) Orotate concentration is not different between hippocampi of anesthetized WT and APP/PS1 mice (6 WT and 6 APP/PS1 hippocampi, p = 0.92). (C) Representative traces of CA1 LFP recordings depict epileptiform spikes in the baseline (Bsl) and after TERI i.c.v. injection in anesthetized (1.5% isoflurane) APP/PS1 mice. Scale bars: 1 mV, 1 min. (D) TERI i.c.v. injection caused a significant reduction in the frequency of epileptiform spikes from 7.6 ± 1.4 to 3.8 ± 0.95 per min (n = 9 mice, p = 0.004). (E) VEH i.c.v. injections did not affect the frequency of epileptiform spikes (n = 7 mice, p = 0.58). (F) On average, the TERI-treated group displayed 50.7% ± 7.2% reduction (p = 0.0003) in the frequency of epileptiform spikes (the same data as in D and E). Wilcoxon matched pairs (D and E) and Mann-Whitney nonparametric two-tailed test (B and F) were used for the analysis. ^∗∗^p < 0.01; ^∗∗∗^p < 0.001. The error bars represent SEM. See also Figures S10C and S10D.

Finally, we asked whether lowering MFR set points can present an effective way to suppress CA1 hyperexcitability under anesthesia in fAD mice. Our recent work has uncovered the mitochondrial DHODH enzyme as a regulator of MFR set points and Ca^2+^ buffering by mitochondria during spiking activity (Styr et al., 2019). Namely, we showed that DHODH inhibition by TERI negatively regulates CA1 MFR set points and suppresses CA1 hyperexcitability in a genetic model of Dravet syndrome (Styr et al., 2019), one of the most intractable and severe forms of childhood epilepsy. To test whether DHODH inhibition suppresses CA1 hyperexcitability associated with fAD mutations, we first compared the dose response of TERI on CA3-CA1 synaptic transmission in hippocampal slices prepared from WT and APP/PS1 mice. The IC_50_ of TERI on fEPSP amplitude was not different between genotypes (Figure 7A; 17.6 μM in WT and 19.1 μM in APP/PS1), indicating that DHODH activity is not altered by fAD mutations. Moreover, a negative regulation of MFR set points by TERI was preserved in APP/PS1 networks *ex vivo* (Figures S10C and S10D), suggesting that fAD mutations do not impair homeostatic regulation of MFR set point by DHODH. In addition, the concentration of orotate, the direct product of DHO oxidation by DHODH, was similar in the hippocampi of anesthetized WT and APP/PS1 mice (Figure 7B), confirming that DHODH activity is not affected by fAD mutations. Based on these results, we decided to inhibit cerebral DHODH *in vivo* and test how it affects pathological CA1 activity in anesthetized APP/PS1 mice. Because TERI does not cross the blood-brain barrier efficiently (Vidal-Jordana et al., 2015), we used intracerebroventricular (i.c.v.) infusion of TERI (27 μg in 1 μL) versus vehicle (VEH; 1 μL) (Styr et al., 2019) to test the effect of DHODH inhibition on aberrant CA1 activity under anesthesia in APP/PS1 mice. On average, i.c.v. infusion of TERI caused a ∼50% decrease in the rate of epileptiform high-voltage CA1 spikes in comparison to baseline recordings (Figures 7C, 7D, and 7F). In contrast, a similar amount of VEH did not affect aberrant CA1 activity (Figures 7E and 7F). These results indicate that cerebral DHODH inhibition dampens CA1 hyperexcitability under anesthesia in APP/PS1 mice.

## Discussion

Understanding how neural circuits are dysregulated at the early presymptomatic stages of AD is of utmost importance for detection, treatment, and perhaps even prevention of the disease. Electrophysiological and imaging studies provide compelling evidence of network-wide dysfunctions of neural circuits in patients with AD and animal models (Frere and Slutsky, 2018; Palop and Mucke, 2016; Vossel et al., 2017; Zott et al., 2018). Moreover, impairments of homeostatic control have been associated with a wide range of brain disorders, including neurodevelopmental (Mullins et al., 2016; Ramocki and Zoghbi, 2008) and psychiatric (Kavalali and Monteggia, 2020) disorders. However, whether failures in firing rate homeostasis are gated by specific brain states and contribute to the disease progression remain unknown. Our results provide converging evidence on disrupted neural signatures of two low-arousal brain states, anesthesia and sleep, by fAD mutations at early stages in the disease progression.

Our study demonstrates that during active wakefulness, APP/PS1 mice are no different than WT mice in terms of CA1 firing rate distributions, CA3-CA1 synaptic transmission, and short-term synaptic plasticity, before the onset of hippocampus-dependent memory decline. The similarity of CA1 MFRs between WT and APP/PS1 mice in active wakefulness was confirmed by two independent recording methods with single-cell resolution: Ca^2+^ microendoscopy and single-unit electrophysiology. However, pronounced dysregulations of CA1 circuits were exposed by NREM sleep in fAD mice. Specifically, the down-regulation of CA1 MFR by NREM sleep was disrupted in fAD mice. If MFR fluctuates between stable homeostatic set-points across sleep-wake cycle, the observed MFR dysregulation during NREM sleep may be caused by fAD-related impairment of downward MFR homeostasis (Figure 6) that is typically induced during extended sleep periods (Torrado Pacheco et al., 2021). Alternatively, dysregulation of MFRs during NREM sleep may result from the impairment of set-point establishment mechanisms. In this case, all the compensatory mechanisms may act in reference to this pathological set-point value, being detrimental for hippocampal functioning. The mechanisms leading to a pathological set point may be associated with abnormal changes in the neuromodulatory tone, which is typically diminished during wake-NREM transitions for the major neurotransmitters and neuromodulators, including acetylcholine, noradrenaline, histamine, orexins, dopamine, serotonin (Brown et al., 2012), and adenosine (Peng et al., 2020). Notably, cholinergic (Mesulam, 2013) and noradrenergic (Grudzien et al., 2007) dysfunctions are evident in mild cognitive impairment and patients with early-stage AD. Further studies may shed some light on malfunctions in neuromodulatory signaling pathway(s) that underlie dyshomeostasis of CA1 firing rates by NREM sleep in the presymptomatic AD stages.

Importantly, our data show that the observed local dysregulation of CA1 MFR set points during NREM sleep precedes global deterioration of SWA, an indicator of AD pathology at early stages of the disease (Lucey et al., 2019). Thus, the loss of natural brakes on spontaneous spiking activity during NREM sleep in local hippocampal networks may represent the earliest state-dependent homeostatic failure induced by fAD mutations. The homeostatic dysregulation of MFR set points during NREM sleep may underlie subclinical epileptiform activity, detected during sleep in a fraction of human patients with AD (Brown et al., 2018; Lam et al., 2017; Vossel et al., 2016) and in fAD models (Brown et al., 2018; Kam et al., 2016). How AD-related changes in synaptic and intrinsic neuronal properties (Frere and Slutsky, 2018; Palop and Mucke, 2016; Selkoe and Hardy, 2016; Styr and Slutsky, 2018; Zott et al., 2018) are altered by brain states and cause state-dependent dysregulation of activity set points remains to be discovered.

The results of this work, taken together with previous studies on the reciprocal relationship between neuronal activity and AD-related pathology (Ju et al., 2014; Wang and Holtzman, 2020), point to a complex role of NREM sleep in AD progression. On the one hand, accumulated evidence suggests that sleep mitigates AD-related pathology. Extracellular Aβ levels are regulated by spiking and synaptic activity, being decreased by activity suppression (Bero et al., 2012; Cirrito et al., 2005; Dolev et al., 2013; Kamenetz et al., 2003). Interstitial Aβ and tau levels are decreased by sleep and increased by sleep deprivation (Holth et al., 2019; Kang et al., 2009; Lucey et al., 2018; Xie et al., 2013). Because sleep is associated with a homeostatic decrease of MFRs (Miyawaki and Diba, 2016; Senzai et al., 2019; Vyazovskiy et al., 2009) and homeostatic down-scaling of excitatory synapses (de Vivo et al., 2017; Diering et al., 2017), this may be the cause of physiological fluctuations in the interstitial Aβ levels during the sleep-wake cycle (Roh et al., 2012). On the other hand, our results point to homeostatic dysregulation of firing rates by NREM sleep in local CA1 hippocampal circuitry of APP/PS1 mice. This may be a potential reason for disrupted Aβ fluctuations during the sleep-wake cycle in APP/PS1 mice (Roh et al., 2012). Because homeostatic dysregulation of CA1 MFRs precedes global sleep disturbances, rescue of these homeostatic mechanisms during sleep may provide an early opportunity to prevent or slow down deterioration of sleep patterns and subsequent accumulation of pathological AD hallmarks.

In addition to sleep, physiological regulation of firing rates was impaired during general anesthesia in fAD mice. General anesthesia is defined as a drug-induced reversible behavioral state that is associated with unconsciousness, amnesia, analgesia, and akinesia, with concomitant homeostasis of vital physiological functions (Brown et al., 2011). Despite decades of research, experimental evidence is lacking on how anesthetic drugs affect homeostatic regulation of central neural networks. Utilizing an *ex vivo* system that enables long-term spike recordings from the same neurons in neural networks, we were able to show that distinct anesthetic drugs lower MFR set point in WT neural networks. However, the homeostatic response of APP/PS1 neurons to isoflurane was completely disrupted. Instead of a stable suppression, MFR was rapidly compensated and returned within 1–2 h to the original set point despite the presence of isoflurane. This disruption of homeostatic set-point regulation by anesthetic drug in hippocampal networks *ex vivo*, disconnected from other brain structures, is translated to profound dysregulation of hippocampal activity under general anesthesia in fAD mice *in vivo*. At the level of the CA1 population, the hallmarks of anesthesia-induced changes in neuronal activity—suppression of firing rates and reduction in the number of discriminable network microstates—were impaired across anesthetic depth. As a result, hyperactivity and hypersynchrony of CA1 networks were exposed by anesthesia in several fAD models, including APP/PS1, 5XFAD, and APP-KI. In light of these results, anesthesia may account for some of the causes of neuronal hyperexcitability described in earlier two-photon Ca^2+^ imaging studies performed under isoflurane in the APP/PS1 model (Busche et al., 2008, 2012, 2019; Grienberger et al., 2012).

It is worth mentioning that only a few studies performed state-dependent analysis of aberrant brain activity in fAD mouse models. Among those, epileptiform activity was completely absent from wakeful behavior in Tg2576 mice (Kam et al., 2016). Interestingly, J20 mice showed fewer aberrant spikes during exploratory behavior associated with increased gamma activity (Hanson et al., 2020; Verret et al., 2012). At the single-unit level, APP-KI (*App*^NL−G-F^) mice displayed normal CA1 MFRs during active wakefulness, while exploring a familiar environment (Jun et al., 2020). In addition, normal MFRs were detected in the CA3 and dentate gyrus of APP/PS1 mice during exploratory behavior (Rechnitz et al., 2021). Even if some abnormal brain activity can be revealed under specific conditions in active wakefulness, our results strongly suggest a robust increase in excitability by general anesthetics as a common feature of different fAD models.

The anesthetic state and natural sleep share many neurobiological features, yet they are two distinct states (Akeju and Brown, 2017). Therefore, different molecular mechanisms may underlie dysregulation of neural correlates of NREM sleep and general anesthesia by fAD mutations. In this study, we found that fAD mutations do not impair DHODH enzymatic activity under anesthesia; thus, inhibition of cerebral DHODH by TERI effectively suppressed MFR set point *ex vivo* and pathological CA1 hyperexcitability in anesthetized APP/PS1 mice *in vivo*. These results suggest that identification of signaling pathways that (1) negatively regulate MFR set points and (2) are resilient to fAD mutations may present a therapeutic opportunity to prevent hippocampal hyperexcitability during low-arousal brain states. Notably, DHODH is primarily expressed in neurons in the brain (Schaefer et al., 2010) and regulates MFR via mitochondrial Ca^2+^ (Styr et al., 2019). Because dysregulation of mitochondrial Ca^2+^ has been recently implicated at the more advanced disease stages in the APP/PS1 model and in patients with AD (Calvo-Rodriguez et al., 2020), DHODH inhibitors may be powerful blockers of activity-dependent mitochondrial Ca^2+^ overload (Styr et al., 2019). Interestingly, the concentration of orotate, the direct product of DHODH enzymatic reaction, is decreased during NREM sleep in the cortex of WT mice (Bourdon et al., 2018). How fAD mutations affect cellular-molecular mechanisms underlying MFR down-regulation by anesthesia versus NREM sleep remains an important challenge for future investigations.

In summary, our data demonstrate that anesthesia and sleep, two distinct altered states of low arousal, expose CA1 network dysfunctions that are hidden during active wakefulness. The observation that early hippocampal pathophysiology emerges only during specific brain states provides perspectives on the circuit-level basis of AD-related vulnerability. Based on our data, we propose that sleep fragmentation and decrease in sleep time, detected at later AD stages, may present a compensatory response to CA1 neuronal hyperexcitability during NREM sleep in an effort to maintain daily MFR homeostasis. Whether impaired neural correlates of anesthesia and sleep constitute an early pathophysiological hallmark of presymptomatic AD remains to be determined by future human studies.

### Limitations of the study

Some limitations should be considered when interpreting our results. First, the homeostatic nature of MFR downregulationduring NREM sleep is still awaiting further experimental support. Second, although we demonstrate that fAD mutations impair upward MFR homeostasis in response to a chronic perturbation *ex vivo*, these results should be validated *in vivo*. Finally, although the DHODH inhibitor presents an opportunity to suppress AD-related epileptiform spikes under anesthesia, its role in sleep disturbances remains to be established.

## STAR★Methods

### Key resources table

**Table:** 

REAGENT or RESOURCE	SOURCE	IDENTIFIER
**Bacterial and virus strains**		

AAV5-CaMKIIα-GCaMP6f	University of North Carolina Vector Core	Custom preparation

**Chemicals, peptides, and recombinant proteins**		

Isoflurane	Sigma Aldrich	CAS: 26675-46-7
Ketamine hydrochloride	Sigma Aldrich	CAS: 1867-66-9
Xylazine hydrochloride	Sigma Aldrich	CAS: 23076-35-9
Teriflunomide	Tocris Bioscience	Cat# 5069;CAS: 108605-62-5
R-Baclofen	Tocris Bioscience	Cat# 0796;CAS: 69308-37-8
Gabazine	Abcam	Cat# ab120042;CAS: 104104-50-9
Buprenorphine	Sigma Aldrich	CAS: 52485-79-7

**Experimental models: Organisms/strains**		

Mouse: C57BL/6J	The Jackson Laboratory	JAX: 000664
Mouse: APP/PS1 hemizygotes on a C57BL/6J-congenic background	The Jackson Laboratory	JAX: 005864
Mouse: homozygous *App*^*NL-G-F*^ knock-in mice	Saito et al. (2014)	N/A
Mouse: heterozygous 5xFAD mice	Oakley et al. (2006)	N/A

**Software and algorithms**		

Inscopix Data Processing Software 1.2.1	Inscopix	https://www.inscopix.com
MATLAB	MathWorks	MATLAB 2019
GraphPad Prism 8	GraphPad	https://www.graphpad.com/scientific-software/prism/
CNMF-E	Zhou et al., 2018	https://github.com/zhoupc/CNMF_E
AccuSleep – sleep scoring software	(Barger et al., 2019)	https://github.com/zekebarger/AccuSleep
KlustaKwik – spike sorting software	Kadir et al. (2014)	http://klustakwik.sourceforge.net/
Cell Explorer – cell type characterization	(Petersen et al., 2021)	https://cellexplorer.org/
Custom MATLAB© code used to produce the analysis results	This paper	https://doi.org/10.5281/zenodo.5779904

**Other**		

nVistaHD 2.0 or nVista 3.0 miniscopes + DAQs	Inscopix	https://www.inscopix.com
GRIN Lens Probe	Inscopix	1050-002176
Glass tube	vitroCom	CV1518-B-003
Custom coverslips	Thermo scientific	25 mm #0 (round)
Baseplate	Inscopix	1050-004201
Baseplate cover	Inscopix	1050-002193
Coated stainless steel wire	A-M system	791400
Male connectors	Harwin Inc.	M20-9990545
Amplifier	custom-made amplifier	N/A
Digitizer	Digidata 1440A by Molecular Devices	https://www.moleculardevices.com/
Monochrome camera	GigE Vision, Basler AG	https://www.baslerweb.com/en/
Microdrive	Custom-built by Rogat Enterprises	N/A
Electronic interface board (EIB)	Custom-built by Rogat Enterprises	N/A
Recording wires (tetrodes)	California Fine Wire	Model:M283720 size: 17mm, Platinum 10% Iridium
Head-stage connector	Omnetics	NPD-18-DD-GS
Gold pins	Neuralynx	Large EIB pins
Electrophysiology acquisition system	Tucker-Davis Technologies	PZ5, RZ5D
Electrophysiology head stages	Tucker-Davis Technologies	LP16CH-Z
Bone cement	C&B-metabond	https://www.parkell.com/c-b-metabond_3
MEA2100-Systems	Multi Channel Systems	https://www.multichannelsystems.com/products/mea2100-systems
MEA2100-Mini-Systems	Multi Channel Systems	https://www.multichannelsystems.com/products/mea2100-mini-systems
SomnoSuite Low-Flow Anesthesia System	Kent Scientific Corporation	https://www.kentscientific.com/products/somnosuite/

### Resource availability

#### Lead contact

Further information and requests for resources should be directed to and will be fulfilled by the lead contact, Inna Slutsky (islutsky@tauex.tau.ac.il).

#### Materials availability

This study did not generate new unique reagents.

### Experimental model and subject details

All animal experiments were approved by the Tel Aviv University Committee on Animal Care. *In vivo* experiments were performed on 4-5 month old male and female APP/PS1 (APP_Swe_/PS1ΔE9) hemizygotes (Stock No. https://www.jax.org/strain/005864, The Jackson Laboratory) and their wild-type littermates. *In vivo* electrophysiological experiments were performed on the following 4-5 month old transgenic mice and their littermates: (1) APP/PS1 mice, (2) homozygous *App*^*NL-G-F*^ knock-in mice (Saito et al., 2014) (APP-KI), co-expressing Swedish (KM670/671NL), Iberian (I716F) and Arctic (E693G) mutations, and (3) heterozygous 5xFAD mice https://www.nature.com/articles/s41593-020-0624-8 - ref-CR12(Oakley et al., 2006) (provided by Dr. Dan Frenkel lab), co-overexpressing mutant forms of human APP associated with the Swedish, the Florida (I716V) and the London (V717I) mutations. Only male mice were used for memory and sleep experiments, while both male and female mice were used for electrophysiological and Ca^2+^ imaging recordings under anesthesia. All mice were on a C57BL/6J-congenic background. All animals were kept in a normal light/dark cycle (12h/12h, lights on at 7AM) with free access to food and water. Mutant and wild-type mice were housed together for behavioral, biochemical and EEG/EMG experiments. Mice for Ca^2+^ imaging / single-unit recordings were singly housed after the microendoscopy / microdrive implantation.

### Method details

#### General surgical procedures

In all the surgical procedures, the mice were anaesthetized with 5% isoflurane by volume for induction, injected i.p. with ketamine/xylazine (100 mg/kg ketamine and 8 mg/kg xylazine), head fixed to a stereotaxic apparatus (David Kopf instruments) and then maintained anesthetized by continuous isoflurane (1.5%) inhalation throughout the surgical procedure. Eye ointment was used to protect the mice eyes (Duratears, Vetmarket) and body temperature was recorded and maintained by a heating pad (FHC, DC temperature controller) at 34°C throughout the surgery. At the beginning of each surgical procedure the mice were injected sub cutaneous with Carprofen (5mg/kg) to reduce inflammation and pain. The mice were then allowed to recover in their home cage for at least 1-2 week before the subsequent surgical procedure or experiment began. When studying the effects of distinct anesthetics on physiological CA1 properties, individual anesthetics were used as specified in the figure legends.

For ICV injections, a small hole was drilled in the skull above the left lateral ventricle (0.7 mm posterior, 1.2 mm lateral to bregma), and a 5mm guide cannula was slowly inserted into the ventricle and fixed to the skull by dental cement (C&B Metabond, Parkell). The guide cannula was sealed with a 5 mm sterile metal bar to prevent CSF leakage and possible infections. 1-2 weeks after the surgery, the mice received i.c.v. injections using a 10 μl syringe (Hamilton). The mice were injected with 1 μl containing 27 μg of Teriflunomide (TERI), dissolved in DMSO or vehicle (VEH, 1 μl of DMSO) with speed of 0.15 μl/min (Nano Jet stereotaxic syringe pump).

#### LFP/fEPSP recordings

Small diameter holes were drilled in the skull at the position of the recording and stimulating electrodes. The recording electrode (bipolar, PFA-coated stainless steel; 0.127 mm diameter) was slowly lowered through the cortex into the CA1 *stratum radiatum* (2.06 mm posterior to bregma; 1.5 mm mediolateral, ML; 1.5 mm dorsoventral from bregma, DV), and the stimulating electrode (bipolar stainless steel; 0.127 mm diameter) was slowly lowered through the cortex into the Schaffer Collateral (SC, 2.54 mm posterior to bregma; 2.75 mm ML; 2.2 mm DV). Ground electrode was screwed to the skull above the cerebellum. Test stimuli of 50-100 μA were delivered to the SC at 0.06 Hz to verify the proper location of the electrodes and to estimate the stability of the signal. The electrodes were firmly fixed to the skull by dental cement (C&B Metabond, Parkell) and dental acrylic. Operated mice recovered in their home cage for at least a week following electrodes implantation. For evoked fEPSP recordings in CA3-CA1 synapses in awake mice (Figures 1J and 1K), mice were habituated for a few days to the experimental device that was composed from a running wheel and metal bar in which the mice were head fixed into throughout the experiment with a screw. Following habituation, the mice were head fixed to the experimental device, and CA3-CA1 fEPSP measurements were taken 30 minutes after.

Extracellular field potentials and electromyograms were amplified x100 using a custom-made amplifier, band-pass filtered between 0.1 Hz and 4 KHz, and digitized by Digidata 1440A at 56 kHz sampling rate (Molecular Devices). Data were analysed using Clampfit 10.7 (Molecular devices) for fEPSP's or custom MATLAB functions (MathWorks).

#### EEG/EMG recordings

EEG screws were placed over the frontal and parietal cortices. Ground and reference screw electrodes were placed above the cerebellum. Neck muscle electrodes were implanted bilaterally, and bipolar referencing was used for EMG. A custom-made EEG/EMG connector was fixed on the skull with dental cement for sleep recordings.

#### Surgical procedure for Ca^2+^ imaging

The surgical procedures were previously described (Ziv et al., 2013). First, 500 nL of the viral vector AAV5-CaMKII-GCaMP6f (prepared by University of North Carolina Vector Core) was injected into the CA1 pyramidal layer at the following coordinates: −2.1 mm AP, −1.5 mm ML, and −1.3 mm DV to bregma. The skin was sutured and disinfected using Betadine solution. Two weeks after virus injection, a glass guide tube was implanted directly above CA1. For this, a trephine drill was used to remove a circular part of the skull located postero-lateral to the viral injection site, and the dura, cortex and the hippocampal commissures above the CA1 were removed by suction with a 29 gauge blunt needle while constantly washing the exposed tissue with sterile PBSx1. A glass guide tube was then implanted above the CA1 *stratum pyramidal*. A recording electrode (bipolar stainless steel; 0.127 mm diameter) was slowly lowered through a hole that was drilled adjacent to the guide tube into CA1 *stratum radiatum* (1 mm DV). The space between the skull and the optical guide tube was sealed using a low toxicity silicone adhesive (Kwik-Sil, WPI surgical instruments) and the remaining exposed area of the skull was covered with dental cement and dental acrylic. A metal bar was added to the posterior aspect of the construct in order to head fix the animal when needed.

#### Anesthetic depth analysis

Anesthetic depth was modulated by isoflurane level: 1% for moderate anesthesia and 1.5% for deep anesthesia. For vital physiological measurements, mice were placed in an induction chamber connected to isoflurane vaporizer (Isotec 5), gas flow rate was turned on to 0.8 LPM and the vaporizer was set at 5% isoflurane. When mouse’ breathing slowed down and became rhythmic, it was moved to physiological monitoring system (75-1500 Harvard Apparatus) and isoflurane level was reduced gradually and slowly to either 1.0% or 1.5%. Respiratory rate was monitored and analyzed by the physiological monitoring system. Temperature was also maintained by the device at 37°C throughout the procedure. No changes in respiratory rate was found between all the genotypes used in the paper at 1.0% and 1.5% isoflurane (Figure S9). Analysis of burst-suppression ratio (BSR) is described in ‘quantification and statistical analysis’ section.

#### Ca^2+^ imaging in behaving and anesthetized mice

For time-lapse imaging in behaving mice we used an integrated miniature fluorescence microscope (nVistaHD 2.0 or nVista 3.0, Inscopix) as previously described (Ziv et al., 2013). At least two weeks after the glass guide tube implantation, we inserted a microendoscope consisting of a metal guide cannula (∼3.1mm length, 1.8mm outer diameter) and a single gradient refractive index lens (4.0mm length, 1.0mm diameter) into the implanted glass tube and examined Ca^2+^ indicator expression in the operated mice (Inscopix data acquisition software, Inscopix). We selected for further imaging only those mice that exhibited homogenous GCaMP6f expression throughout the field of view, without signs of injury or inflammation (∼85% mice passed the selection criteria). For the selected mice, we then affixed the microendoscope within the glass guide tube using ultraviolet-curing adhesive (Flow-It A3, Pentron). Next, we attached the miniature microscope’s magnetic base plate to the dental acrylic surface with the ultraviolet-curing adhesive. A day later, the mice were habituated for 4-5 days to freely explore a 50 x 50cm open-field, with the miniature microscope. Before the beginning of each session, the open-field was thoroughly cleaned with a 70% Ethanol solution. To record mouse behavior, we used an overhead monochrome camera (GigE Vision, Basler AG), which we synchronized with the miniature microscope. Behavioral analysis was performed with ToxTrac software (Rodriguez et al., 2018). Ca^2+^ imaging was performed at 10 Hz. Imaging sessions consisted of 5-15-min-long trials, while the inter-trial interval was ≥ 15 min.

For Ca^2+^ imaging sessions during different types of anesthesia, the mice were head-fixed to a stereotactic device before the application of anesthesia and their body temperature was maintained by a temperature controller (FHC, 40-90-8D). LFP activity in the CA1 *stratum radiatum* was recorded throughout the duration of anesthesia and Ca^2+^ measurements were taken once a stable LFP pattern was observed, 60 minutes following anesthesia induction. For the inhalatory anesthetic isoflurane, experiments were performed under 1.0% and 1.5% isoflurane, combined with oxygen (100%). Ketamine (100 mg/kg), supplemented with xylazine (8 mg/kg), were injected i.p. Medetomidine (0.3 mg/kg) was injected i.p and stopped using the synthetic α_2_ adrenergic receptor antagonist Antipamezole (1mg/kg, i.p). The drugs were diluted with sterile PBS before injection.

For Ca^2+^ imaging during sleep-wake cycle, two stainless-steel wires were inserted to either side of neck muscles, and referenced bipolarly, to measure EMG activity. Imaging analysis was performed during extended (≥5 min with less than 30 sec interruption by the other state) bouts of active wakefulness or NREM sleep based on LFP/EMG recordings during 6 hours of the light cycle (10 AM – 4 PM). 5 kHz noise in the LFP/EMG recordings generated by electronic focus of the nVista3 microscope was filtered out by a band-pass filter (1-300 Hz). Mice were habituated to the recording chamber for several days before the experiment.

#### Single-unit surgery and data acquisition

Mice were implanted with a costume-made microdrive (custom printed circuit board and drive by Rogat, Carmiel, Israel) along with neck muscle electrodes implanted for electromyography (EMG). The microdrive contained a moveable assembly of 4 tetrodes (17-μm, Platinum 10% Iridium, California Fine Wire) and was connected to the recording setup via an Omnetics headstage connector (Connector Corporation, Minneapolis MN, USA). Two holes were drilled in the skull: one in the frontal bone plate for a screw serving as ground; the second hole for the electrodes implanted in the parietal cortex (1.94 mm posterior of bregma; 1-1.2 mm medial lateral axis; 1-1.2 mm dorsal ventral axis). After 7 days of monitored recovery, subsequent downward movements of the microdrive were made in 25- to 50-μm increments over 24-hr intervals until approaching the CA1 pyramidal cell layer, recognized by the appearance of multiple high-amplitude units and spontaneous ripple events. At the end of the experiment, a small electrolytic lesion was made (30 μA for 20 sec) under anesthesia. Two days after, histology procedure was performed to verify electrodes location as described (Weiss et al., 2017).

Animals were recorded during the first half of the light cycle in a familiar environment (home cage). Raw data were sampled at 24 KHz using a Neurophysiology Workstation (RZ5D base processor and PZ5 NeuroDigitizer amplifier, Tucker-Davis Technologies Inc).

#### Detection of pathological spikes in fAD models

High-voltage pathological spikes were detected by setting a threshold 10 z-scores above and below the mean voltage during the entire recording and accepted only if their peak-to-peak value within 30 ms was greater than 10 z-scores. A dead-time of 50 ms was set to assure the same spike was not counted twice. Subsequently, all spikes were manually inspected and approved.

#### Scoring of vigilance stages

Vigilance states were manually scored in 5-s epochs based on visual inspection of frontal EEG or CA1 LFP and EMG as previously described (Barger et al., 2019). Wakefulness was defined by low-amplitude, high-frequency EEG/LFP activity, recorded in the frontal lobe, and high EMG activity. Wakefulness was further divided to states of active wake (exploration, grooming, eating) and quiet wake based on video and EMG recording. NREM sleep was defined by high-amplitude, low-frequency EEG/LFP, and reduced EMG tone. REM sleep was defined by low-amplitude, high-frequency EEG/LFP, dominated by theta activity, and with flat EMG. States transitions and artifacts (<3% of recording time) were excluded from further analysis. Spectrograms were constructed by Fourier frequency transformation (1 second bins) of the EEG signal.

#### MEA

Postnatal hippocampal cultures were plated on MEA plates containing 120 titanium nitride (TiN) electrodes, in addition to 4 internal reference and 4 ground electrodes (Styr et al., 2019). Each electrode has a diameter of 30 μm and electrodes are arranged in a 12X12 grid (sparing 6 electrodes in each corner), spaced 100-200 μm apart on average [Multi Channel Systems (MCS), 120MEA200/30iR-Ti]. Data acquisition was done in 3-weeks-old cultures using a standard MEA2100-Systems and MEA2100-mini-Systems (MCS) with a hardware filter cut-off of 3.3 kHz and sampling rate of 10 kHz per electrode. Recordings were carried out under constant 37°C and 5% CO2 levels.

#### Electrophysiology in slices

Acute hippocampal slices (coronal, 400 μm) were prepared from WT and APP/PS1 mice. Slices were transferred to a submerged recovery chamber at 32°C containing oxygenated (95% O2 and 5% CO2) artificial cerebrospinal fluid (ACSF) for 1h before the experiment. The ACSF contained, in mM: NaCl, 125; KCl, 2.5; CaCl2, 1.2; MgCl2, 1.2; NaHCO3, 25; NaH2PO4, 1.25; glucose, 25. fEPSPs were recorded in acute hippocampal slices with a glass pipette containing Tyrode solution (1 – 2 MΩ) from synapses in the CA1 stratum radiatum using a MultiClamp700B amplifier (Molecular Devices). Stimulation of the Shaffer Collateral (SC) pathway was delivered through a glass suction electrode (10 – 20 μm tip) filled with Tyrode. Data were analyzed using pClamp10 (Molecular Devices).

#### Protein extracts and ELISA for Aβ

After transcardial perfusion with cold PBS hippocampus were dissected, snap-frozen in liquid nitrogen, and stored at −80°C until use. Proteins from both hippocampus of 5 month old APP/PS1 and APP-KI mice were sequentially extracted in a 2-step procedure. Tissue (∼0.2 mg/mL wet weight) was homogenized using a mechanical homogenizer in 50 mMTris-HCl, pH 8.0, 150 mM NaCl, complete proteinase inhibitor cocktail (Roche) and 10mg/mL Pepstatin A, centrifuged at 100,000 g for 1 hour at 4°C, and supernatants containing the soluble fraction were collected, stored at −80°C, and used for quantification of soluble Aβ. Pellets were resuspended, incubated on ice for 1h and sonicated in 6 M guanidine-HCl, 50 mM Tris-HCl, pH 7.4, centrifuged at 100,000 g for 1 h at 4°C, and supernatants containing membranous fraction and insoluble aggregates were collected, stored at −80°C, and used for quantification of insoluble Aβ. Protein quantification was performed using Bradford method. The levels of soluble Aβ40 and Aβ42 in mice hippocampus extracts were detected using sandwich ELISA kits (Wako, Japan), according to the manufacturer's instructions. Concentrations of soluble and insoluble Aβ are expressed in pM per μg of total soluble or insoluble protein, respectively.

#### Spatial working memory test

Spatial working memory was examined using a continuous variation of the T-maze (Wood et al., Neuron, 2000). In sum, mice were required to traverse the central arm of a delta-shaped maze and alternate between left and right turns during subsequent trials. Correct trials were reinforced with a drop of condensed milk at the edge of the selected side arm. After entering one of the side arms, retracing was prevented by hinged doors such that mice could initialize a new trial only by returning to the base of the central arm via a connecting arm. At the base, mice were confined for a specified delay before the door to the central arm was opened and a new trial commenced. The first trial of each session contained a reward in both side arms of the maze. Prior to the onset of training, mice were habituated to the apparatus for 15 m a day for 5 consecutive days. During training, mice performed 10 trials of the task with a 10 s delay. During testing, the delay varied between days from 10 to 180 s. To facilitate learning and assure consistent motivation when performing the task, food intake of mice was restricted such that their weight was kept at 85 – 90% relative to *ad libitum* feeding. Success rate was defined as the percentage of correct alternations relative to the number of trials.

#### Contextual fear conditioning test

Contextual fear conditioning (CFC) was performed in a 25.5 x 25.5 x 36 cm chamber with electric grid floor. For the conditioning session, the mice were placed in the CFC apparatus for 2.5 min, and then a pure tone (2.9 kHz) was introduced for 20 sec, followed by a 0.6 mA foot shock for 2 sec. Another tone and shock were introduced again after 1 min, and then, 30 sec after the second shock, the mice were returned to their home cages. The context in which the mice received foot shock consisted of green lighting, white noise, vanilla scent and square perspex walls. To test contextual fear memory, mice were placed back in the familiar context for 5 min. The mice were later placed in a novel context for 2.5 min, consisted of white lighting, rum scent and round walls. The apparatus was cleaned between every session using 70% Ethanol and Virusolve. Automatic freezing detection of the recorded videos was conducted using the EthoVision software.

#### Histological verifications

To check the expression of AAV5-CaMKII-GCaMP6f in the CA1 and the precision of the injection location and micro endoscope implantation site, we used 2-photon microscope (LSM 7 MP, Zeiss) to image the pyramidal layer in hippocampal slices. Chameleon Ti:Sapphire laser system with a 80 MHz repetition rate was used to excite the sample. The excitation wavelength was 920 nm. Emission light was filtered by 500 – 550 nm band-pass filter. Three-four weeks after injection of the virus or at the end of the behavioral experiments, we perfused mice with phosphate-buffered saline (PBS) followed by cold 4% paraformaldehyde (PFA). We then removed the perfused brains and kept them in PFA solution for 24h. 70-μm coronal slices were obtained from the perfused brains using a Leica VT1200 vibrating microtome and stored in PBS for further imaging. Validation of the injection site, implanted micro endoscope location and viral expression was obtained by imaging in 2-photon microscope.

#### Metabolic profiling

For determining orotate concentration in the hippocampus of WT and APP/PS1 mice, mice were anesthetized by 1.5% isoflurane and hippocampi were dissected and then homogenized by Bullet Blender homogenizer (Next Advance) at 4°C with methanol: acetonitrile: water (5:3:2 ratio) solution at 40 mg/ml concentration. Homogenized samples were centrifuged for 15 min at 4°C at 16,000 x g. The supernatants were transferred to glass HPLC vials and stored at −80°C. Metabolic profiling was done using an Ultimate3000 UHPLC system (Dionex, Thermo Scientific) coupled to a Q-Exactive Plus mass spectrometer (Thermo Scientific). Metabolite separation was done using a 49 min gradient of buffer A (95% acetonitrile) and buffer B (50 mM ammonium carbonate, pH 10, 5% acetonitrile) using SeQuant ZIC-pHILIC column (Merck; 150 3 2.1 mm, 5 mm) coupled to a SeQuant ZIC-pHILIC guard column (Merck; 20 3 2.1 mm, 5 mm) with flow rate of 0.1 ml/min. Data were acquired by switching between negative and positive polarity modes using full MS scans. Identification of orotate was done using LCquan software (Thermo Scientific) based on external standards.

### Quantification and statistical analysis

While data collection was not performed blind to the condition of experiment, investigator was blind to these conditions throughout much of the analysis, as spikes/calcium signals were automatically detected and their manual inspection happened without any knowledge of experimental conditions.

#### Single-unit data analysis

Spikes were detected offline as crossings above four STDs of the whitened data (Pachitariu et al., 2016). Spike waveforms (40 samples symmetrical around the peak) were semi-automatically clustered using KlustaKwik (Kadir et al., 2014) followed by manual inspection using Klusters (Hazan et al., 2006). All subsequent data analysis was done using MATLAB (Mathworks, Natick MA). Clusters were defined as well-isolated single units and included in the analysis only if they fulfilled the following criteria: (1) the presence of a refractory period (less than 0.5% of inter-spike intervals <2 ms (Middleton et al., 2018); (2) an isolation distance >10 (Tanaka et al., 2018), (3) MFR >0.05 Hz during active wakefulness; and (4) a stable firing rate during the recording period (Styr et al., 2019). Using the CellExplorer open source software (Petersen et al., 2021), clusters were separated to regularly-spiking and fast-spiking units semi-automatically by inspecting their waveform, autocorrelations and burst index (Royer et al., 2012).

To compare of MFRs between active wakefulness and NREM sleep, we normalized the differences in response magnitudes by the maximum absolute value, to produce an unbiased gain factor (Sela et al., 2020) as follows:

$$\%Gain_{\left(a-b\right)}=\frac{a-b}{\max\left(\left|a\right|,\left|b\right|\right)}\times 100$$

where *a* is the MFR in NREM sleep and *b* is the MFR in active wakefulness. Therefore, a negative gain factor indicates a reduced response during NREM sleep, and vice versa. Gain factors across neurons are summarized as median, and [lower and upper] 95% CIs around the median.

#### Ca^2+^ imaging data analysis

We pre-processed the raw imaging data using a commercial software (Inscopix data processing software, Inscopix). Raw data was spatially down sampled by a factor of 2, cropped to 1200 by 840 μm rectangle, motion corrected and exported as TIFF file. Single neurons and their calcium signals for each data set were extracted using the constrained non-negative matrix factorization algorithm for endoscopic recordings (Zhou et al., 2018) (CNMF-E). CNMF-E algorithm can reliably extract cellular signals from one-photon calcium imaging data sets by parallel denoising, deconvolution and demixing. The maximal diameter of neurons in the imaging plane was set to 13 pixels (gSiz), and the width of the gaussian kernel, which can approximate the average neuron shape was set to 3 pixels (gSig). Besides pre-processing spatial down sampling, no further spatial or temporal down sampling was performed (ssub=1, tsub=1 respectively). Neurons with spatial overlap ratio greater than 0.85, temporal correlation of calcium traces greater than 0.85 and centroid distance less than 1 pixel (dmin) were merged. Region of interest (ROIs) that had a minimum peak-to-noise ratio for a seeding pixel of 8 (min_pnr) and minimum spike size of 5 (smin, when the value is negative, the actual threshold is = |smin∗noiselevel|) were extracted. The foopsi method was used for deconvolution (Pnevmatikakis et al., 2016). These parameters were used for all data sets analyzed in the paper. Due to one-photon background fluorescence fluctuations, motions artifacts and segmented dendrites, some of the ROIs detected by CNMF-E cannot be considered as true cells, but false positive detections. Therefore, we have inspected the registered ROIs spatial footprints and excluded them based on the following exclusion criteria: minimum peak-to-noise ratio of 8, 300 > ROI size > 30 pixels, and minimum circularity estimate of 0.5 (1 equals a perfect circle). Filtered data sets were further manually inspected and verified by the experimenter (∼5-10% cells were excluded per data set). CNMF-E "S" output was used as the inferred spiking activity (firing rate) obtained from the denoised ("C") scaled version of DF ("C_raw"). "Firing rate" of a cell was defined as it summed inferred spiking activity (obtained from the "S" output) throughout the recording session, divided by the recording time. Single-cell and population analysis were performed with custom MATLAB functions.

For network pattern analysis, we created a sequences of inferred spike times per cells that were identified using the CNMF-E algorithm. We projected this data onto a single timeline to produce an ordered time sequence that demonstrates individual cell firing activity. Next, we measured the relative synchronized activity per time bin of 100 ms, defined as:

$${\left(Relative\;Synchronization\right)}_{n}=\;\frac{\sum {\left(Active\;cells\right)}_{n}}{\left(Total\;cells\;detected\right)}\;$$

In which "n" equals to the time bine analyzed. Synchronization vector peaks that were higher than the 90th percentile in exploration sessions, or higher than 0.05 in anesthesia sessions were defined as network bursts. Threshold of 0.05 was chosen for anesthesia sessions since it gave the most reliable network bursts detection based on visual inspection and verification of the data by the experimenter. Similar results were obtained when testing different thresholds (e.g. 0.1 and 0.15, data not shown). We than analyzed the inter-burst interval, number of cells that were active in each network burst, and number of spikes that comprised each network burst. 20 random samples were taken from each mouse for pooled analysis.

#### Burst-suppression ratio analysis

For detecting burst-suppression (deep anesthesia), raw LFP recordings were divided to 500 ms bins and for each bin three parameters were calculated: (1) maximum absolute value, (2) standard deviation, and (3) the first principle component of the spectrogram calculated between 1-100 Hz. Each of these parameters showed a bimodal lognormal distribution and thus were used to classify the bins as periods of bursts or suppression using a gaussian mixture model. Epochs of bursts were merged if the inter-burst interval was less than 2 seconds and were accepted as bursts only if their duration was greater than 2 seconds. These criteria were empirically selected as they increased the algorithm’s robustness. Further, manual scoring revealed that indeed >95% of bursts were longer than 2 seconds. Burst-suppression ratio (BSR) was calculated in ∼1 min bins (52.428 s) as the fraction of time in suppression. Overall, this algorithm demonstrated >92% accuracy when compared to manual scoring of two data sets from different genotypes. Relative delta power was calculated in ∼1 min bins as the power spectral density (PSD) between 1-4 Hz divided by the broadband PSD in 1-100 Hz. This ratio was z-scored and normalized between 0 and 1. Epochs of deep anesthesia were defined as 0.3 < BSR < 0.8 and epochs of moderate anesthesia were defined as BSR < 0.3 and relative delta power > 0.5. These epochs were merged if the inter-epoch interval was less than 1 min and were accepted only if their duration was greater than 1 minute.

#### Microstate clustering

##### t-SNE/WS algorithm

We identified microstates of CA1 hippocampal neuronal population using the un-supervised nonlinear embedding method, t-Distributed Stochastic Neighbor Embedding (Laurens van der Maaten, 2008) (t-SNE) and an image processing algorithm – watershed (WS) combined into t-SNE/WS clustering algorithm. The input for the clustering algorithm was the inferred, de-convoluted spiking patterns based on Ca^2+^ signals provided by the CMNF-E algorithm. The analysis was performed on active frames that included at least 2 co-active neurons as described in an earlier study (Wenzel et al., 2019), with some modifications related to initial dimensionality reduction and perplexity value calculation. For noise reduction, we used an initial dimensionality reduction based on principle component analysis (PCA). The principal components >30^th^ percentile of the derivative of the cumulative explained variance were selected and the data was projected upon them to create a lower dimensional space (post-PCA space). The perplexity parameter for the t-SNE was calculated as $\sqrt{\#\;of\;active\;frames}$ in each experiment. Using the calculated perplexity value and initial dimensionality reduction, t-SNE was applied with 1000 iterations to produce a robust 2D embedding space that could be analyzed and visualized. To create density maps, the embedded points on the 2D map, representing patterns of coactive cells per frame, were smoothed by a Gaussian kernel with a standard deviation equal to 1/60 of the maximum coordinate in the embedded space. To turn patterns of coactive neurons into separated microstates, we used a watershed algorithm (Wenzel et al., 2019) on the density map. A microstate was defined as a region created by the WS algorithm which contains a cluster of similar co-activity patterns based on Ca^2+^ imaging data analyzed in Figure 2. The size of each microstate depends on the frequency of a specific pattern during the recording session. For clustering validation, we used an inter- and intra-cluster correlation score, calculated based on PCA analysis. In order to calculate the number of microstates in a randomized data, we permuted the activity in each frame, so the number of co-active cells in each frame was maintained, while the cells' identity was different.

##### Affinity propagation clustering

We used the affinity propagation cluster (APC) algorithm (Frey and Dueck, 2007) as an additional microstates clustering method. It is an efficient clustering algorithm that takes as inputs the similarities between pairs of observations in the dataset (frames, in our case), and finds exemplars and the observations they represent by exchanging real-valued messages between data points. We used the MATLAB (Mathworks) ‘apcluster’ function made available by the authors. We used the post-PCA correlation between frames as the measure of similarity used by the algorithm. We also used convits = 10 and a preference equal to the median similarity of the dataset.

##### Hierarchical clustering

We used MATLAB's ‘linkage’ function to build a hierarchical bottom-up tree with weighted correlation metric based on the PCA data. ‘Cluster’ function was used to cluster the tree with distance cutoff of 0.75.

#### MEA data analysis

Raw data was filtered, offline, at 200 Hz using a Butterworth high-pass filter. Spikes were then detected, offline, using MC Rack software (MCS) based on a fixed threshold set to between 5-6 standard deviations from mean. Twenty minutes of each hour (that were previously shown to reliably represent the MFR of the entire hour, were used for analysis to reduce processing time and analyzed using custom-written scripts in MATLAB (Mathworks) as previously described (Slomowitz et al., 2015). Channels with unstable (>30% change of MFR) baseline recordings during 3-4 hr prior to a perturbation were excluded from the analysis.

#### Statistical analysis

Error bars shown in the figures represent SEM. All the experiments were repeated at least in three different animals and repeated within the animal at least twice. Statistical significance was assessed by unpaired or paired Student’s *t*-tests, Mann-Whitney *U*-tests, one-way analysis of variance (ANOVA), or two-way ANOVA, where appropriate (multiple comparison tests are specified in figure legends). Normality was assessed using the Shapiro-Wilk test. For non-normal distributions, differences between groups were tested with Wilcoxon signed-rank test for paired data and Mann-Whitney test for unpaired data. Comparison of distributions was performed by Kolmogorov-Smirnov test. Statistical analysis was performed using Prism 8.0 GraphPad. The statistical test, p value and the number of cells / mice that went into the calculation are reported in figure legends. Significance was declared at p < 0.05 and all tests were two-sided.

## Data Availability

Datasets generated during the study will be shared by the lead contact upon request. All original code has been deposited at Zenodo and is publicly available as of the date of publication. DOIs are listed in the key resources table. Any additional information required to reanalyze the data reported in this paper is available from the lead contact upon request.

## Supplemental information

Document S1. Figures S1–S10

Document S2. Article plus supplemental information
