# Supplementary material for: Disrupted neural correlates of anesthesia and sleep reveal early circuit dysfunctions in Alzheimer models
Source: Cell Rep. 2022 Jan 18;38(3):110268. doi: 10.1016/j.celrep.2021.110268 (PMC8789564; doi:10.1016/j.celrep.2021.110268)
Supplement: Document S1. Figures S1–S10 [file mmc1.pdf]

Cell Reports, Volume *38*

Supplemental information

Disrupted neural correlates of anesthesia and sleep reveal early circuit dysfunctions in Alzheimer models

Daniel Zarhin, Refaela Atsmon, Antonella Ruggiero, Halit Baeloha, Shiri Shoob, Oded Scharf, Leore R. Heim, Nadav Buchbinder, Ortal Shinikamin, Ilana Shapira, Boaz Styr, Gabriella Braun, Michal Harel, Anton Sheinin, Nitzan Geva, Yaniv Sela, Takashi Saito, Takaomi Saido, Tamar Geiger, Yuval Nir, Yaniv Ziv, and Inna Slutsky

SUPPLEMENTARY INFORMATION

Disrupted neural correlates of anesthesia and sleep reveal early circuit dysfunctions in Alzheimer models

Daniel Zarhin, Refaela Atsmon, Antonella Ruggiero, Halit Baeloha, Shiri Shoob, Oded Scharf, Leore R. Heim, Nadav Buchbinder, Ortal Shinikamin, Ilana Shapira, Boaz Styr, Gabriella Braun, Michal Harel, Anton Sheinin, Nitzan Geva, Yaniv Sela, Takashi Saito, Takaomi Saido, Tamar Geiger, Yuval Nir, Yaniv Ziv, Inna Slutsky.

This file includes:

Figures S1 to S10

Movies S1 to S6

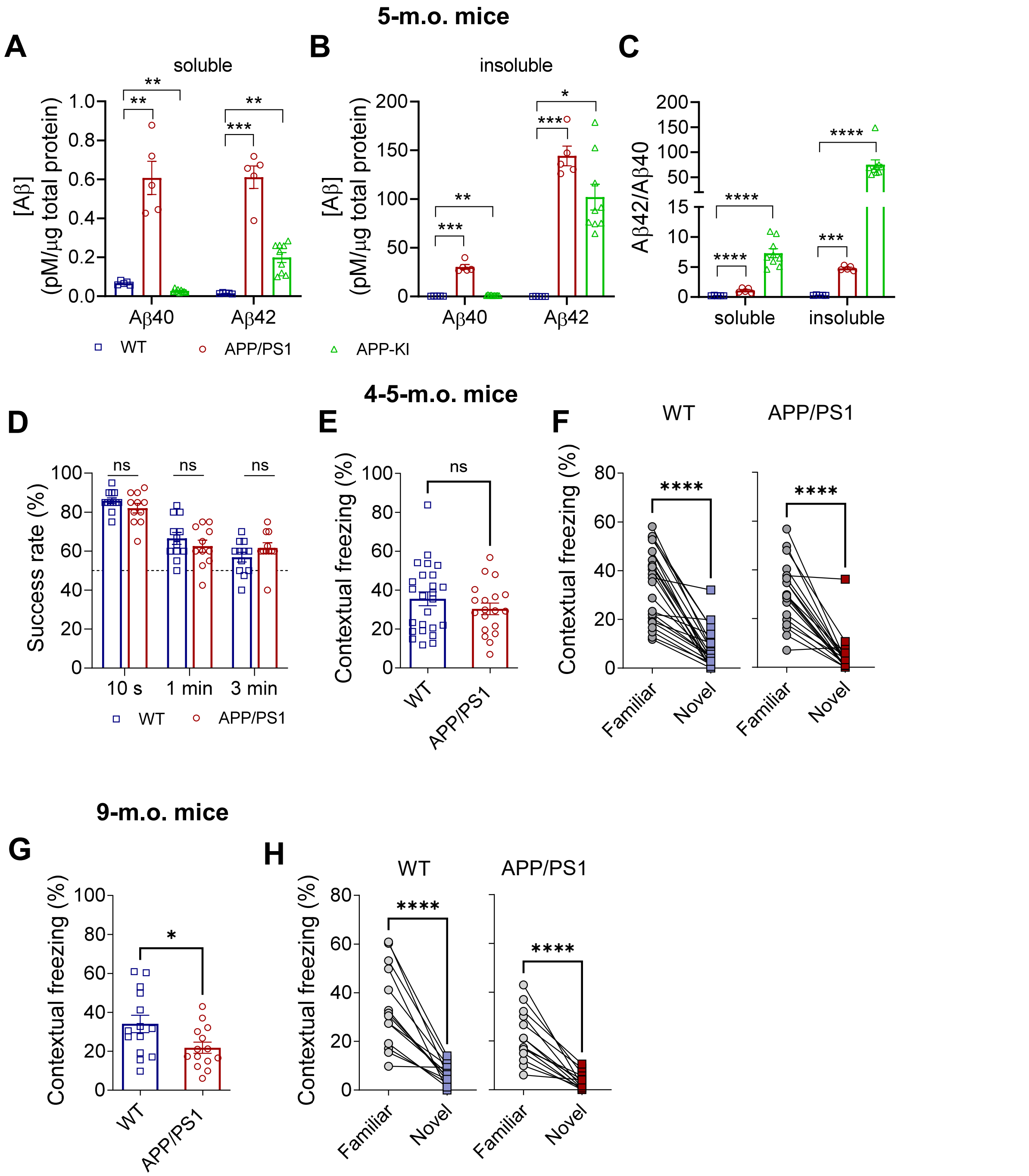

Figure S1. Soluble and insoluble Aβ40 or Aβ42 levels in the hippocampus of WT and cognitively-unimpaired APP/PS1 mice

(**A**) Soluble Aβ40 or Aβ42 levels in WT (n = 5), APP/PS1 (n = 5) and APP-KI (n = 9) 5-m.o. mice.

(**B**) Insoluble Aβ40 or Aβ42 levels in WT (n = 5), APP/PS1 (n = 5) and APP-KI (n = 9) 5-m.o. mice.

(**C**) Aβ42/Aβ40 ratio in soluble and insoluble fractions (the same data as in A,B).

(**D**) Success rate in a continuous variation of the T maze revealed no difference in spatial working memory between WT (n = 12) and APP/PS1 (n = 11) mice (p = 0.69, Two-way ANOVA with Sidak's multiple comparison tests: 10 sec p = 0.66, 1 min p = 0.62, 3 min p = 0.49).

(**E**) No difference in contextual fear memory, tested 1 day after acquisition, between 4-5 m.o. WT and APP/PS1 mice (p = 0.29, WT n = 25, APP/PS1 n=19).

(**F**) Contextual fear memory was specific, reflected by reduction in freezing in the novel context in both, WT and APP/PS1 mice (the same mice as in E).

(**G**) Contextual fear memory was impaired in 9-m.o. APP/PS1 mice in comparison to WT littermates (p = 0.029, WT n = 14, APP/PS1 n = 14).

(**H**) Contextual fear memory was specific, reflected by reduction in freezing in the novel context in both, WT and APP/PS1 mice (paired t-test, the same mice as in G).

Two-way-ANOVA with Dunnett's (A-C) and Sidak (D) multiple comparisons test, un-paired t-test (E,G), paired t-test (F,H) were used for the analysis. *p < 0.05, **p < 0.01, ***p<0.001, ****p<0.0001, ns – non-significant. Error bars represent SEM.

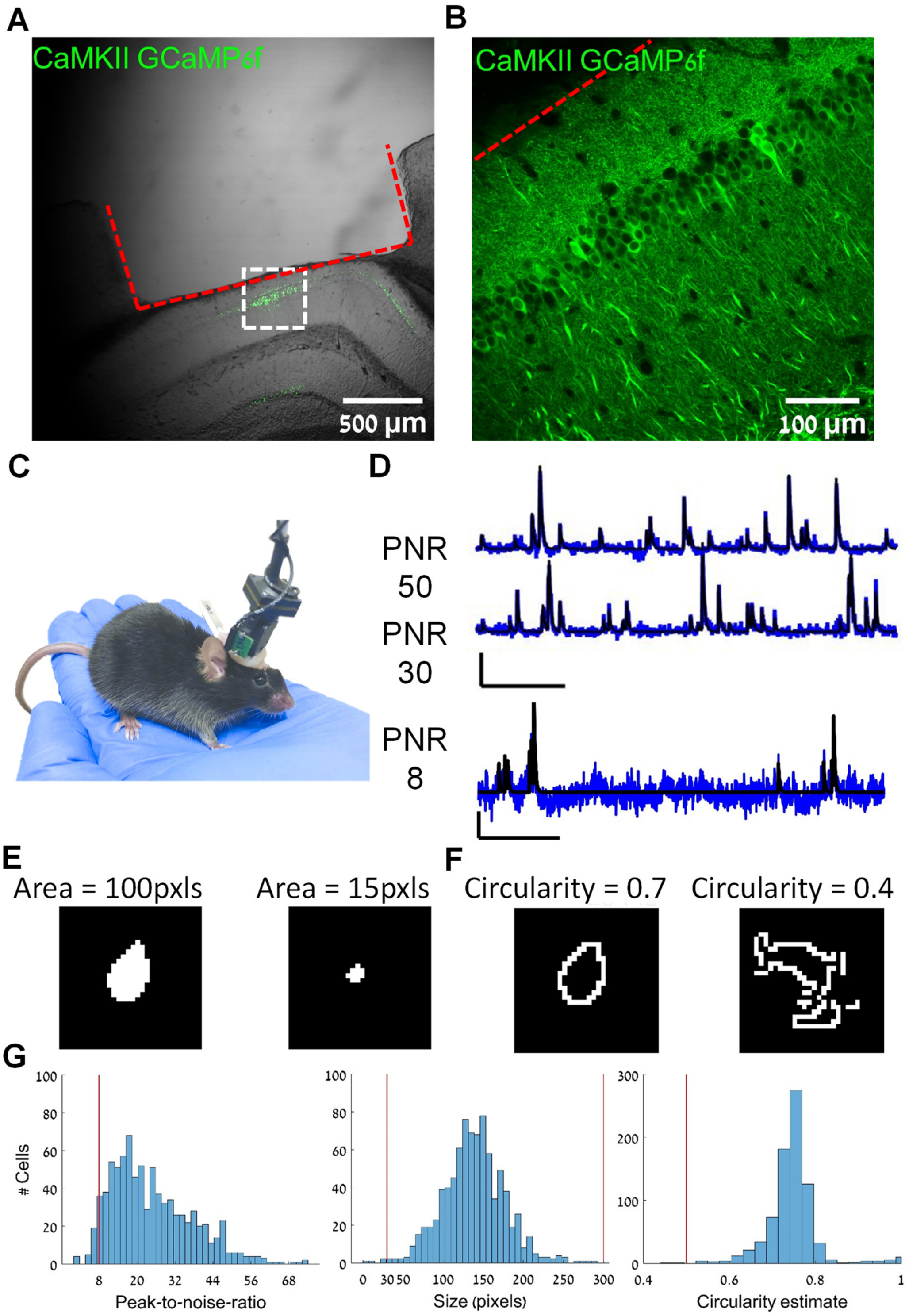

Figure S2. Large-scale Ca^{2+} imaging using wide-field, head-mounted miniaturized fluorescence microscope

(**A**) Coronal section of the cortex and hippocampus showing the area of cortex aspirated (red dotted line) and labeled excitatory CA1 pyramidal neurons expressing the genetically encoded Ca^{2+} sensor GCaMP6f (white dotted square).

(**B**) Enlargement of a, showing individual excitatory CA1 pyramidal neurons expressing the genetically encoded Ca^{2+} sensor GCaMP6f and the approximate location of the GRIN lens used for micro-endoscopic imaging (red dotted line).

(**C**) A mouse carrying a miniaturize fluorescence microscope.

(**D**) Representative traces obtained from the imaging data using the CNMF-E (Zhou et al., 2018) algorithm. Relative scaled fluorescence changes (blue traces) and the denoised versions (black line) of three signals with different peak-to-noise ratios are presented. Scale bars: 1 minute, 20 *z*-scores (two upper traces) and 5 *z*-scores (bottom trace).

(**E-G**) ROIs were limited based on different exclusion criteria: minimum peak-to-noise ratio of 8 (G), ROI size below 30 and above 300 pixels (E,G) and minimum circularity estimate of 0.5 (F,G).

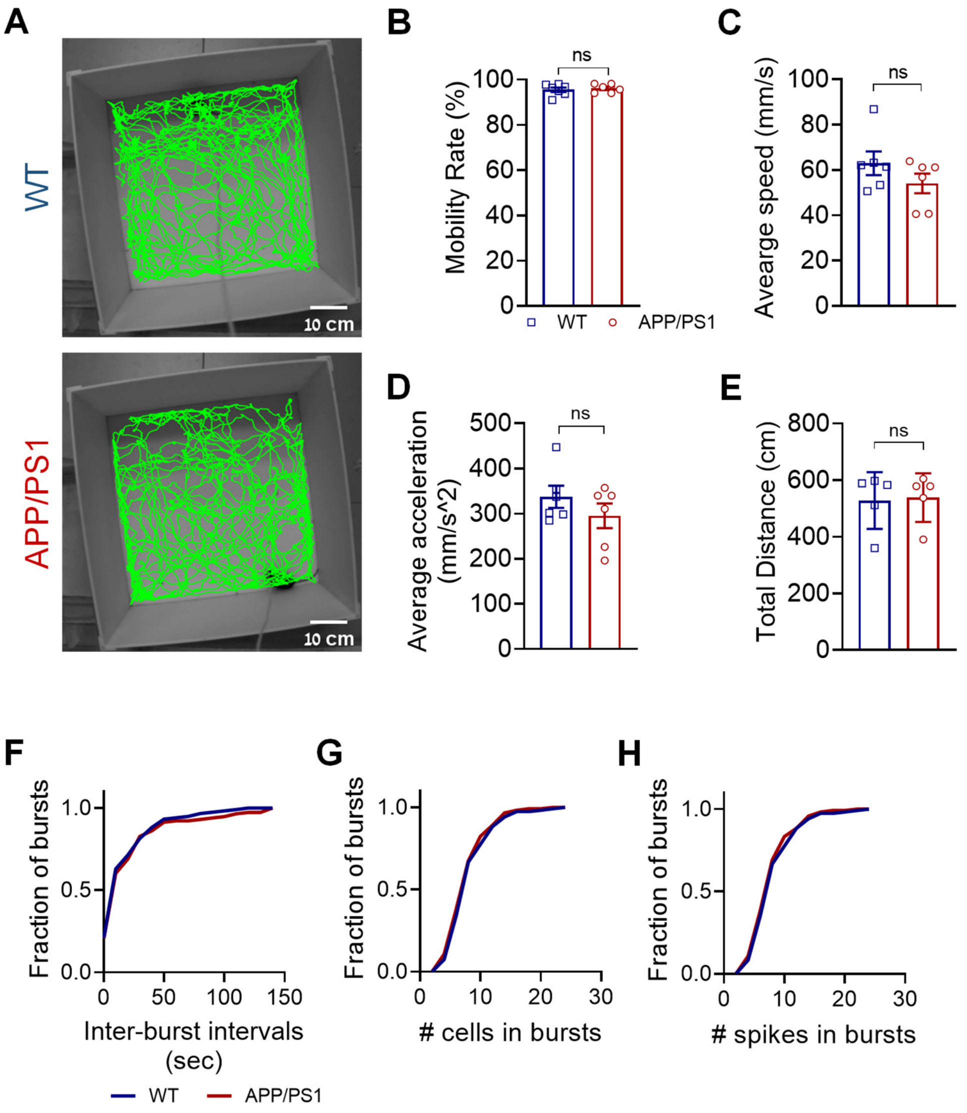

Figure S3. No difference in CA1 neuronal activity pattern and behavioral activity in the open field between WT and APP/PS1 mice

(**A**) Representative traces of WT and APP/PS1 mice locomotor activity while freely exploring a familiar open field for 15 minutes.

(**B-E**) The two experimental groups (6 mice in each group) showed no behavioral differences in the mobility rate (D, p = 0.59), average speed across the imaging session (E, p = 0.60), average acceleration across the imaging session (F, p = 0.94) and total traveled distance (G, p = 0.99). The analysis relates to the mice analyzed in Figure 1C-F. Mann-Whitney U test (D-G) was used for the analysis. ns, non-significant. Error bars represent SEM.

(**F-H**) Patterns of Ca^{2+} transients during free exploration of a familiar environment were similar between WT (6 mice) and APP/PS1 (6 mice). Specifically, no difference was found in the inter-network burst interval (A, p = 0.60), number of cells that participate in each network

burst (B, $p = 0.55$), and the number of spikes that constitute each network burst (C, $p = 0.59$). Relates to the data in Figure 1C-F. Kolmogorov-Smirnov test.

ns, non-significant. Error bars represent SEM.

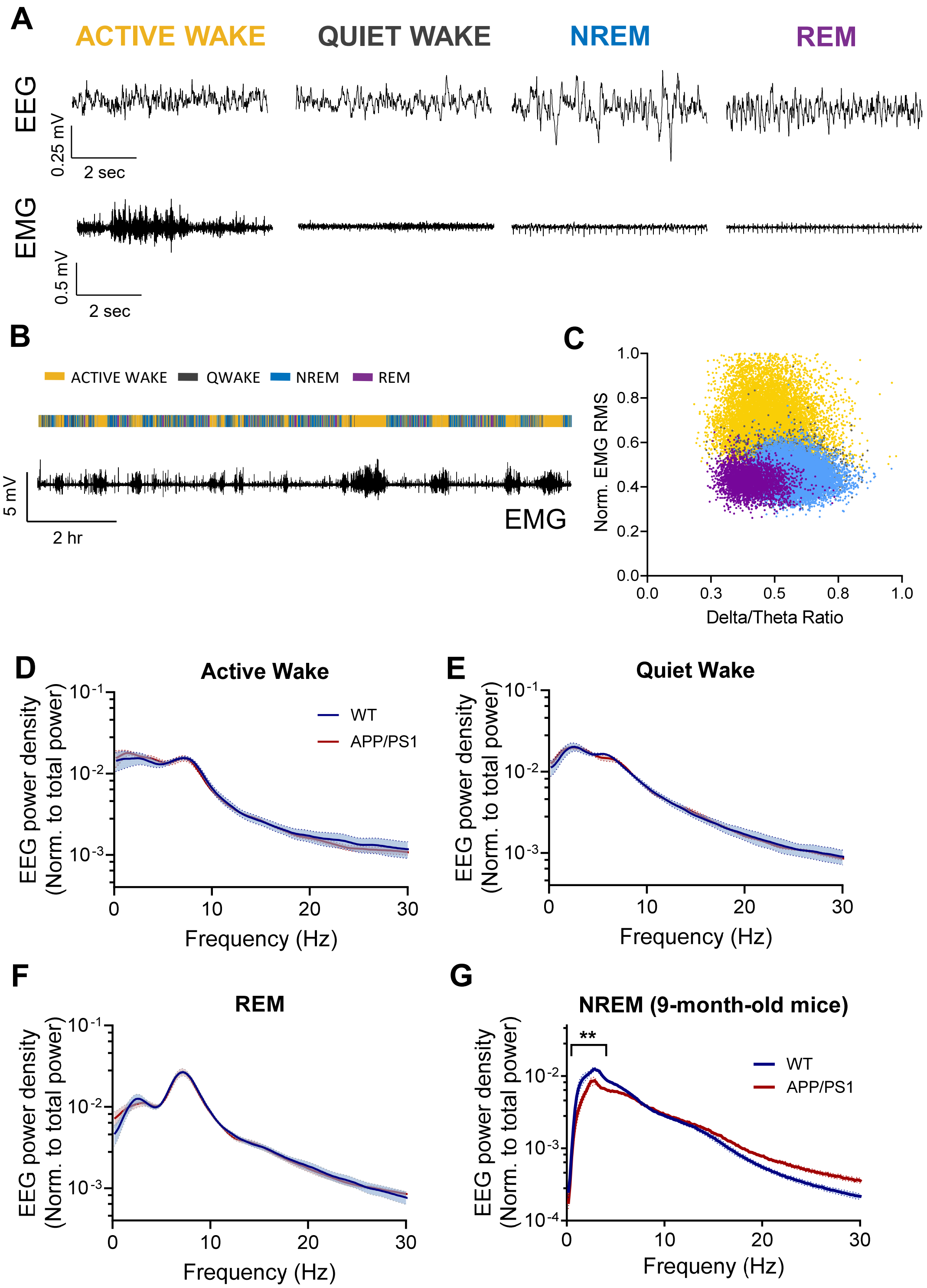

Figure S4. Vigilance states scoring and state-dependent EEG spectral power in WT and APP/PS1 mice

(**A-C**) Representative data from a WT mouse.

(**A**) Representative EEG (top panel) and EMG (bottom panel) traces in different vigilance states: Active WAKE, Quiet WAKE, NREM and REM sleep stages.

(**B**) Representative hypnogram over 12 hours of EEG/EMG recordings during light phase. Brain states are color-coded: yellow - active wake; grey - quite wake, blue – NREM sleep, purple - REM sleep. *Bottom*: EMG trace for the entire 12 hours of recording.

(**C**) Representative scatter plot distribution showing normalized EMG root mean square (*y* axis) versus EEG delta/theta power ratio in 6 sec epochs. Note that NREM and REM sleep are characterized by low EMG levels, and REM sleep and wakefulness are characterized by low delta/theta ratio in the EEG.

(**D-F**) Frontal EEG spectra during active wake (D), quiet wake (E) and REM (F) states in 4-5-month-old WT (n = 5, blue) and APP/PS1 (n = 5, red) mice.

(**G**) Frontal EEG spectra during NREM sleep in 9-month-old WT (n = 5, blue) and APP/PS1 (n = 5, red) mice. Post-hoc comparisons revealed significant decrease in the SWA frequency bins in NREM state (spectral power of 0.5-4 Hz, $p = 0.002$, two-way ANOVA).

Two-way ANOVA with Sidak multiple comparison test (D, F). **$p<0.01$, ns – non significant. Error bars represent SEM.

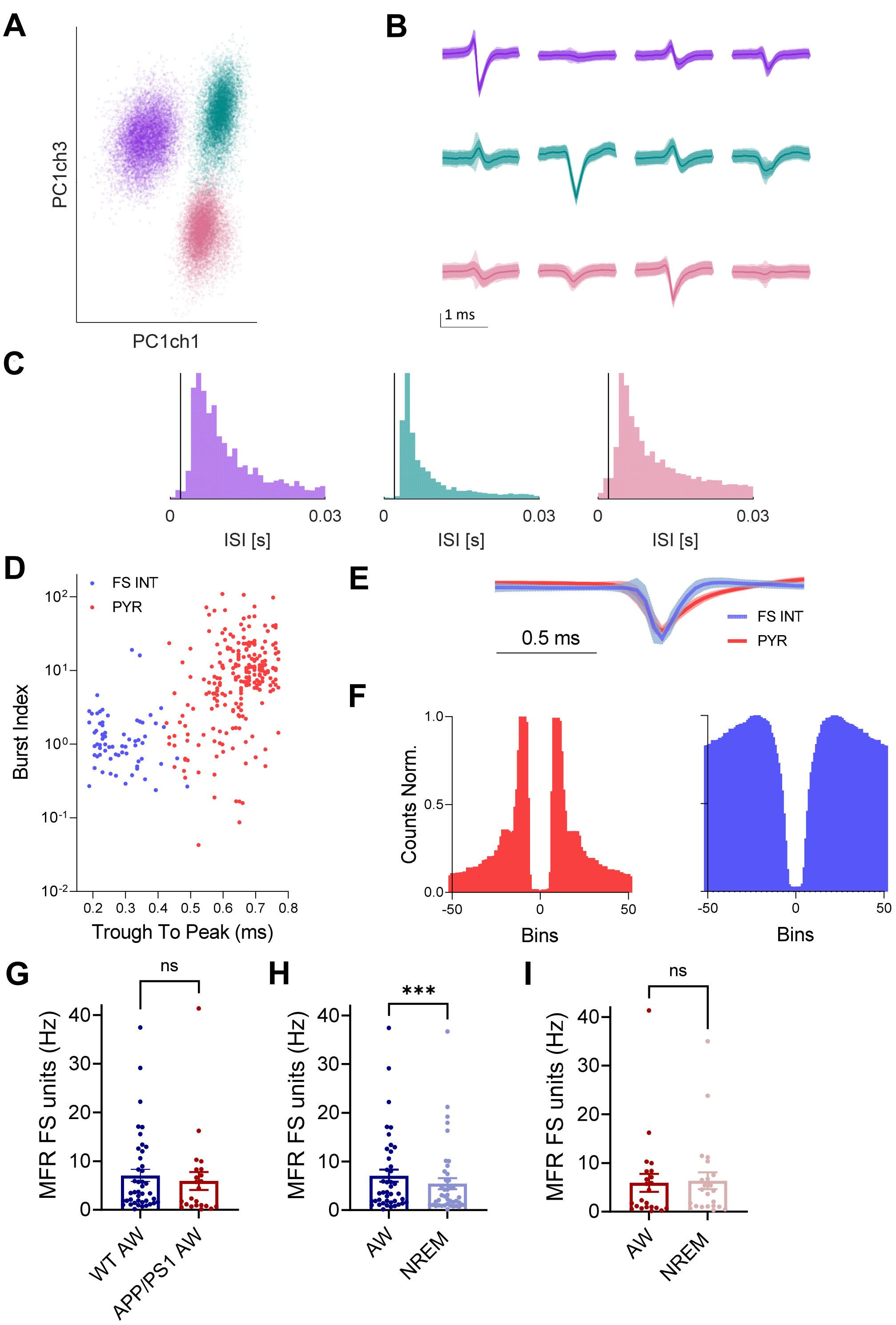

Figure S5. Single-unit recordings in the CA1 of behaving mice (related to Figure 3)

(**A**) Example of three clusters (color coded) recorded from the same tetrode and projected onto the first principle component of channels 1 and 3. Only well isolated clusters (isolation distance > 10) were included in the analysis.

(**B**) Mean ± STD waveform traces of the clusters in (A). Scale bars: 0.05 mV, 1 ms.

(**C**) Inter-spike interval (ISI) histogram of the clusters in (A), vertical line at 2 ms. Only clusters with a well-defined refractory period (less than 0.5% of ISI < 2 ms) were included in the analysis.

(**D**) Separation of regularly-spiking (RS) putative pyramidal neurons (red) and fast-spiking (FS) interneurons (blue) based on trough to peak time and burst index values.

(**E**) Mean ± STD of z-scored waveforms of RS (red) and FS (blue) units.

(**F**) Representative autocorrelogram of an RS unit (*left*, red) and an FS unit (*right*, blue).

(**G**) MFR of CA1 FS neurons was not different (p = 0.22) between WT (7.07 ± 1.25 Hz, 42 single units) and APP/PS1 (5.92 ± 1.84 Hz, 23 single units) during active wakefulness (AW).

(**H**) NREM sleep caused a reduction (p < 0.001) in CA1 MFR of FS interneurons from 7.06 ± 1.25 Hz in AW to 5.9 ± 1.12 Hz in WT mice (42 single units).

(**I**) MFR of CA1 FS interneurons was not different (p=0.33) between AW (5.91 ± 1.84 Hz) and NREM sleep (6.10 ± 1.66 Hz) in APP/PS1 mice (23 single units).

Wilcoxon test (H,I), Mann-Whitney U test (G). ***p<0.001, ns, non-significant. Error bars represent SEM.

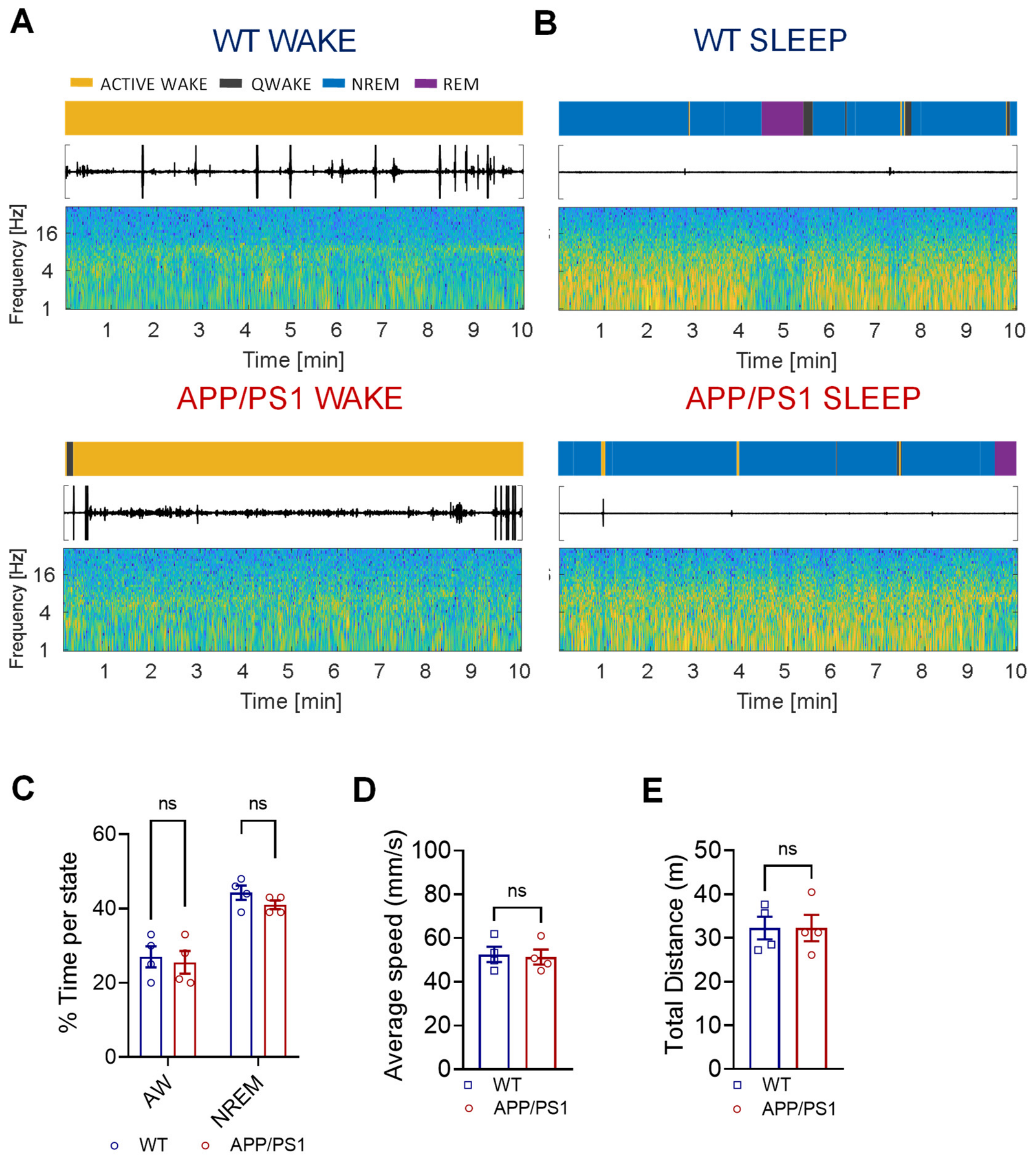

Figure S6. Sleep-wake states analysis in WT and APP/PS1 mice (related to Figure 3)

(**A**-**B**) An example of wake-dense (A) and sleep-dense (B) recordings from a WT (top) and APP/PS1 (bottom) mouse. *Top*: Hypnograms, generated by manual brain state segregation. Brain states are color-coded: yellow - active wake; grey - quiet wake, blue – NREM sleep, purple - REM sleep. *Middle*: EMG traces (scale bar: 1 mV). *Bottom*: Fourier transform-based LFP power spectrograms.

(**C**) Percent of time spent in AW (27.0 ± 2.86 for WT, 25.5 ± 3.07 for APP/PS1) and NREM (44.25 ± 1.93 for WT, 41.0 ± 1.16 for APP/PS1) is not different (p = 0.89 for AW and p = 0.58 for NREM) between WT (n=4) and APP/PS1 (n=4) mice across 6 hours of recording in home cage during light phase.

(**D-E**) No difference in average speed (D, $p = 0.40$, $n = 4$) and total distance (E, $p > 0.9$, $n = 4$) between WT (blue) and APP/PS1 (red) mice across 6 hours of recording in home cage during light phase.

Two-way ANOVA with Sidak's multiple comparisons test (C), Mann-Whitney U test (D-E), ns, non-significant. Error bars represent SEM.

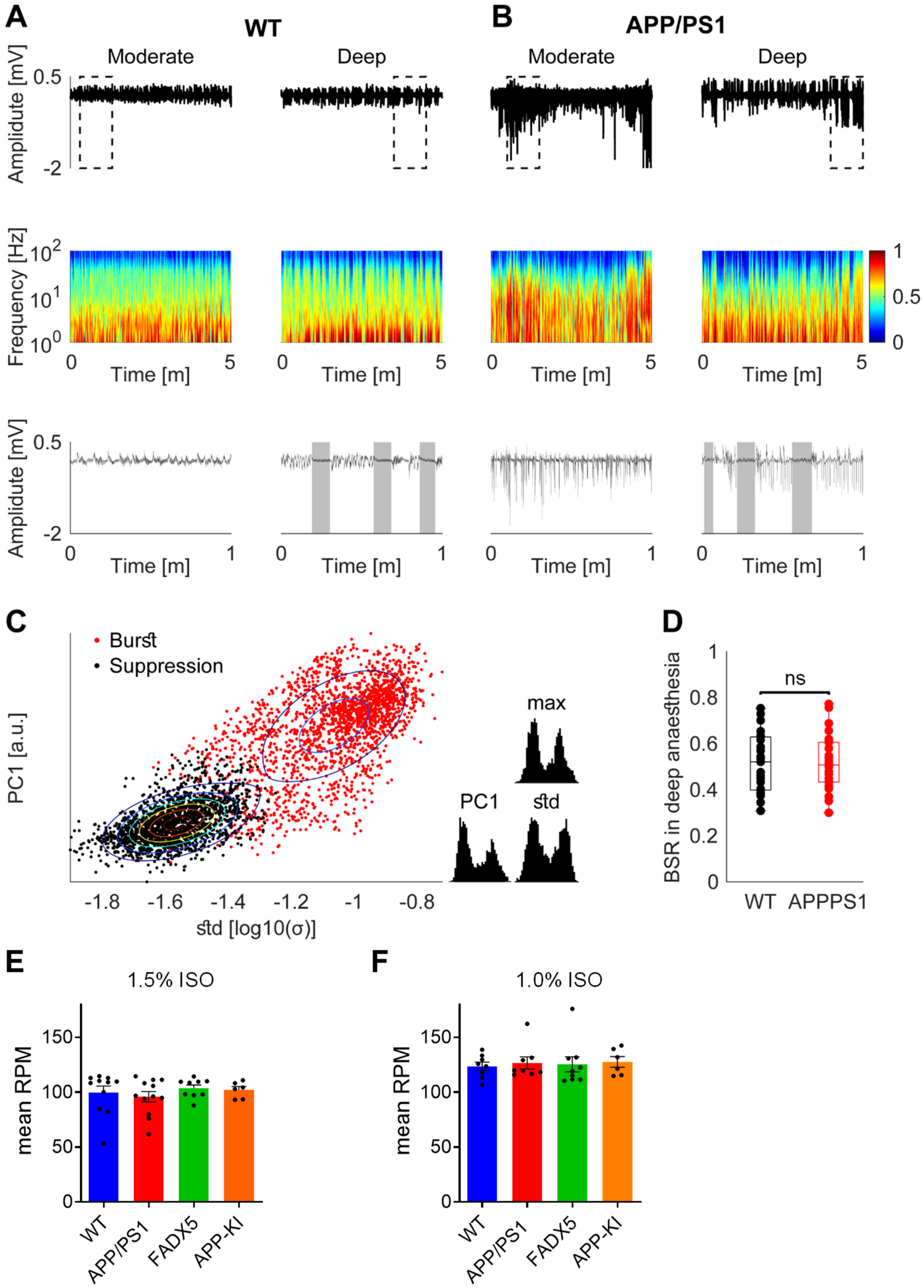

Figure S7. Burst-suppression and abnormal spike detection during different states of anesthesia (related to Figures 4 and 5)

(**A**) From top to bottom: 5 minutes of raw LFP recordings from a representative WT mouse during moderate (*left*) and deep (*right*) anesthesia (top), the corresponding spectrogram

based on short-time Fourier transformation (middle), and an expanded view of the LFP trace marked by a dashed box (bottom). Epochs of suppression are shaded gray.

(**B**) Same as (A) for an APP/PS1 representative mouse.

(**C**) Raw LFP recordings were divided to 500 ms bins and separated to epochs of bursts (red dots) and suppression (black dots) by a gaussian mixture model comprised of three dimensions: standard deviation, first principle component of the spectrogram, and maximum absolute value (not shown). Each of these parameters showed a bimodal lognormal distribution (inset to the right).

(**D**) Comparison of burst-suppression ratio (BSR) in deep anesthesia between WT (27 mice) and APP/PS1 (30 mice). No significant difference was observed (p = 0.98). Mann-Whitney nonparametric test was used for the analysis. ns, non-significant.

(**E**) Respiration rate per minute (RPM) is not significantly different between WT (n = 11) and APP/PS1 (n = 12, P = 0.86), FADx5 (n = 9, P = 0.90) and APP-KI (n = 6, P = 0.98) mice under deep anesthesia (1.5% isoflurane).

(**F**) RPM is not significantly different between WT (n = 8) and APP/PS1 (n = 8, P = 0.96), FADx5 (n = 9, P = 0.99) and APP-KI (n = 6, P = 0.93) mice under moderate anesthesia (1.0% isoflurane). RPM was significantly lower at 1.5% isoflurane in comparison to 1.0% isoflurane for all the groups (p = 0.005 for WT, p = 0.0001 for APP/PS1, p = 0.013 for FADx5, p = 0.020 for APP-KI, two-way-ANOVA with Sidak's multiple comparisons tests).

One-way-ANOVA with Dunnett's multiple comparisons tests was used for the analysis (E,F). Error bars represent SEM.

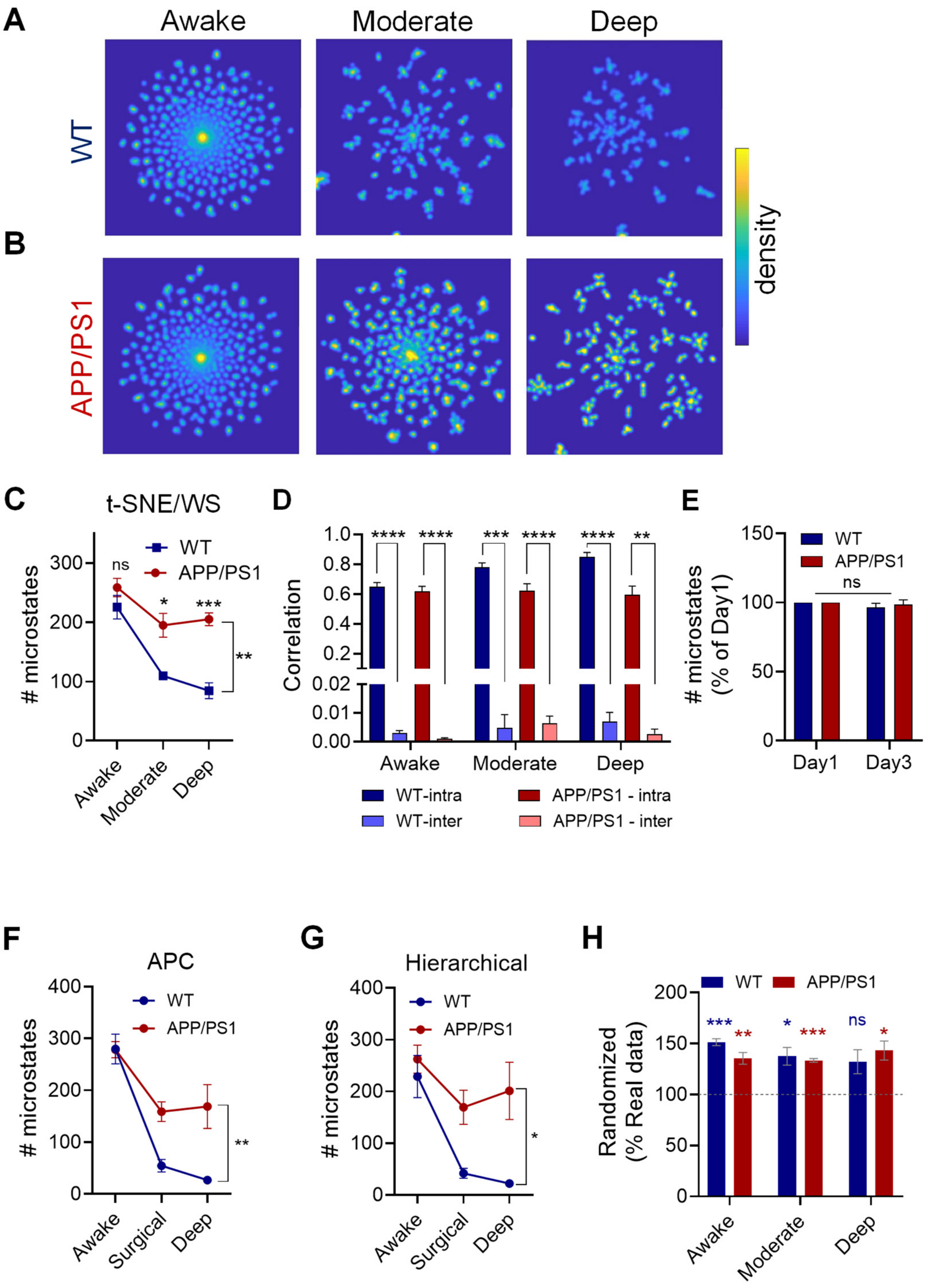

Figure S8. Anesthesia-induced reduction in the number of CA1 microstates is impaired in APP/PS1 mice (Related to Figure 4)

(**A-B**) Representative density maps of microstates, visualized by 2D t-distributed stochastic neighbor embedding (t-SNE) in CA1 circuits of WT and APP/PS1 mice across arousal states: Active Wake (exploration, left panel), moderate anesthesia (central panel), deep anesthesia (right panel) in WT (A) and APP/PS1 (B).

(**C**) t-SNE/ WS (watershed segmentation) clustering analysis in WT (n = 6) and APP/PS1 (n = 6) mice shows that reduction in the mean number of microstates at anesthetic states is impaired in APP/PS1 mice. Note that no difference was observed in wakefulness between WT and APP/PS1 mice ($p = 0.52$).

(**D**) Bar plot of inter- and intra-cluster correlation in the post-PCA space (after data was projected on chosen PCs) for WT and APP/PS1 mice during 3 arousal states; awake, moderate and deep anesthesia.

(**E**) Number of microstates normalized to the number of microstates in day 1 for WT (n=4) and APP/PS1 (n=4) mice during exploration at 2 days interval.

(**F-G**) Number of microstates determined by affinity propagation clustering (APC, F) and hierarchical clustering (G) in WT (n = 6) and APP/PS1 (n = 7) mice across arousal states show that reduction in the mean number of microstates at anesthetic states is impaired in APP/PS1 mice.

(**H**) Number of microstates determined by t-SNE/WS on 100 randomized datasets derived from within-frame shuffling of recorded data. In 5 out of 6 experimental groups, the number of generated microstates was significantly higher in the randomized compared to recorded data (normalized to recorded data).

Two-way ANOVA with Sidak's multiple comparison test were used for the inter-group analysis (C,E-H) and two-way ANOVA with Tukey's multiple comparisons test (D) were used for analysis. *$p<0.05$, **$p<0.01$, ***$p<0.001$, ****$p < 0.0001$. ns – non-significant. Error bars represent SEM.

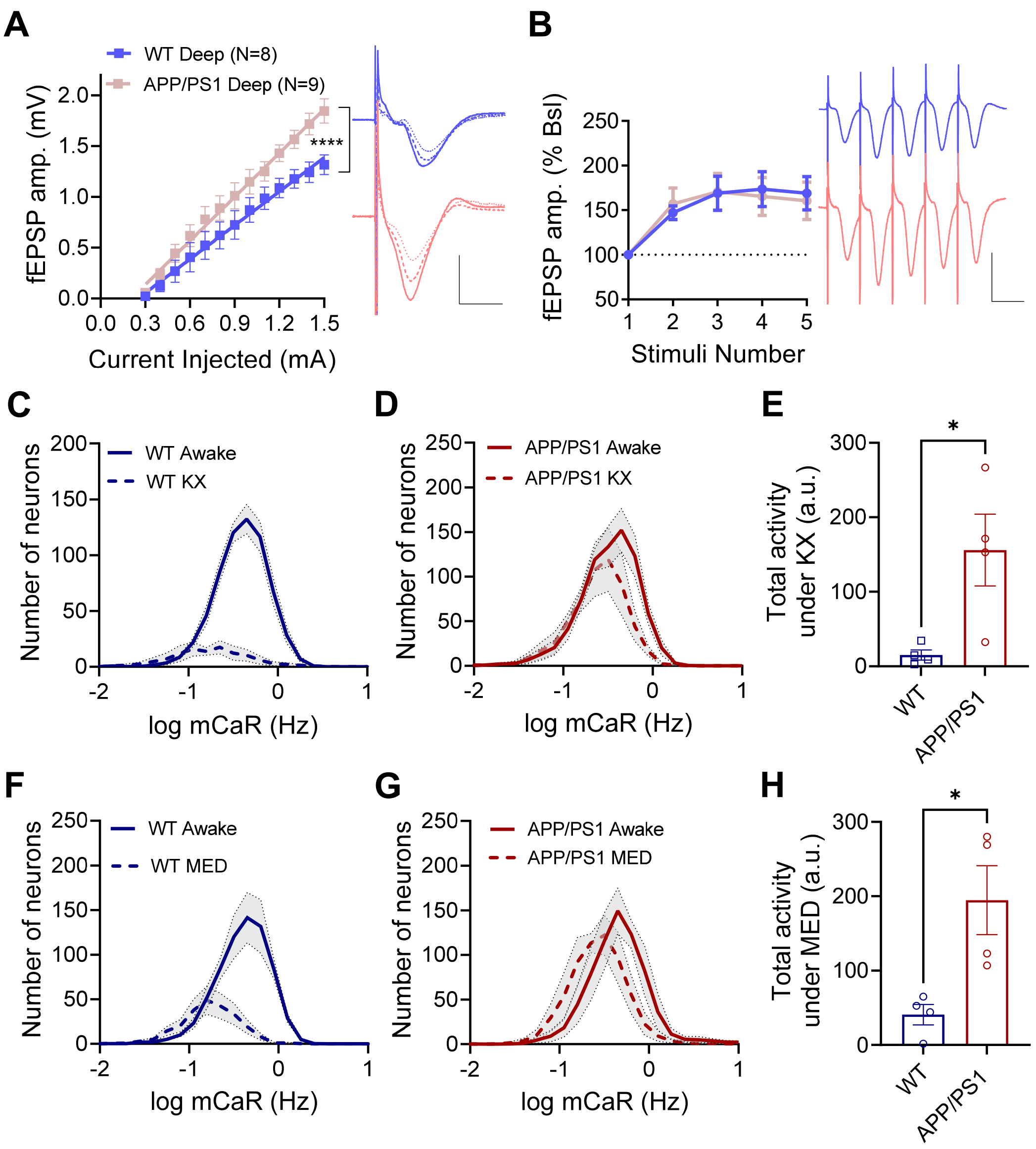

Figure S9. Impairment of neuronal inhibition in APP/PS1 mice by distinct anesthetic drugs (Related to Figure 4)

(**A**) *Left*: Response of fEPSP amplitude recorded in CA1 *stratum radiatum* to increased current injection in the ipsilateral Schaffer Collaterals shows increased input-output slope in isoflurane-anesthetized (1.5% isoflurane) APP/PS1 (9 mice) versus WT (8 mice). *Right*: representative traces of fEPSP of WT and APP/PS1 evoked by 60, 90,120 μA stimulation. Scale bars: 10 ms, 1 mV.

(**B**) *Left*: fEPSP amplitude normalized to the first response during a burst stimulation (five stimuli, 50 Hz) shows similar level of synaptic facilitation between isoflurane-anesthetized (1.5% isoflurane) WT and APP/PS1 ($p = 0.42$). *Right*: representative traces of fEPSP evoked by five stimuli at 50 Hz. Scale bars: 20 ms, 1 mV.

(**C-D**) Effect of Ketamine (100 mg/Kg, i.p.) combined with Xylazine (8 mg/Kg, i.p.) (KX) anesthesia on average mCaR distribution in CA1 of WT (5 mice, C) and APP/PS1 (4 mice, D) groups.

(**E**) Total activity was 6.8-fold higher under KX in APP/PS1 mice (the same data as C-D).

(**F-G**) Effect of Medetomidine (MED, 0.3 mg/Kg, i.p.) anesthetic on average mCaR distribution in CA1 of WT (4 mice, F) and APP/PS1 (4 mice, G) groups of mice.

(**H**) Total activity was 4.3-fold higher under MED in APP/PS1 mice (the same data as F-G).

Two-way-ANOVA with Sidak's multiple comparisons test (A,B) and Mann-Whitney U test (E,H) was used for the analysis. *$p<0.05$, ****$p < 0.0001$. Error bars represent SEM.

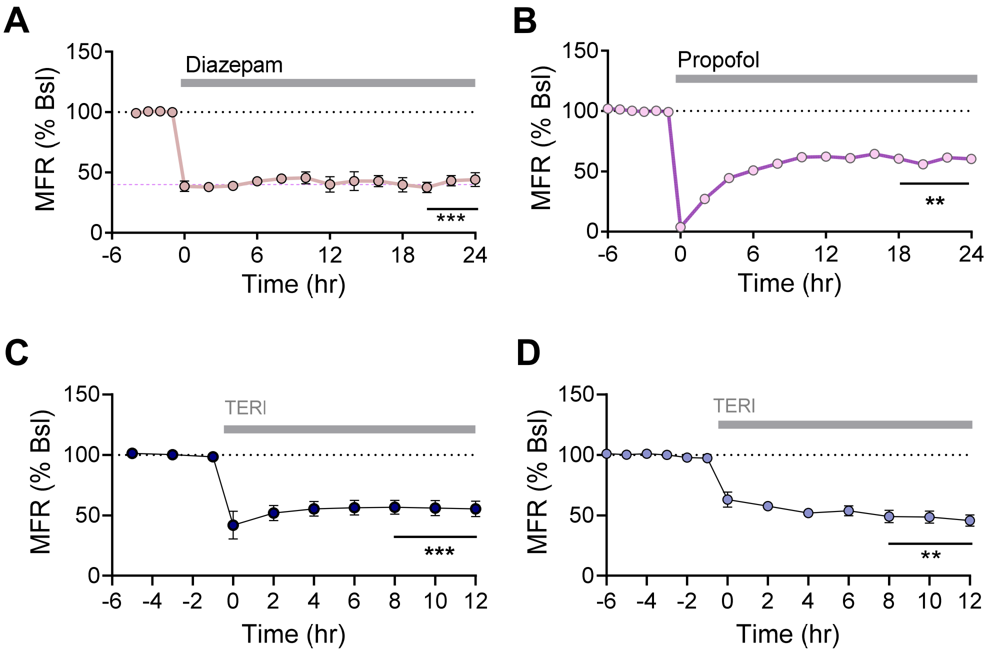

Figure S10. Modulation of MFR set points by anesthetics and DHODH inhibitor (Related to Figure 6)

(**A**) Diazepam (5 μM) stably reduces MFR by ~56% in WT neural networks (5 experiments, 333 channels).

(**B**) Propofol (5 μM) stably reduces MFR by ~40% in WT neural networks (3 experiments, 195 channels).

(**C-D**) Teriflunomide (TERI, 100 μM) stably inhibits MFR by ~45% in WT (A, 7 experiments, 423 channels) and by ~51% in APP/PS1 (B, 3 experiments, 230 channels) neural networks.

Paired t-test for the last 4 hours of a perturbation versus baseline (A-D), $^{**}p<0.01$, $^{***}p<0.001$. Error bars represent SEM.
